# Benchmarking the utility of dry-electrode electroencephalography for clinical trials

**DOI:** 10.1038/s41598-025-18184-7

**Published:** 2025-09-29

**Authors:** Joseph Paillard, Philipp Bomatter, Laura Dubreuil-Vall, Jörg Felix Hipp, David Johannes Hawellek

**Affiliations:** 1 Roche Pharma Research and Early Development, Neuroscience and Rare Diseases, Roche Innovation Center Basel, F. Hoffmann-La Roche Ltd., Basel, Switzerland; 2 Inria, Université Paris-Saclay, Paris, France; 3 Institute for Adaptive and Neural Computation, School of Informatics, The University of Edinburgh, Edinburgh, UK

**Keywords:** Dry-electrode EEG, Clinical trials, Biomarkers, Neurology, Neuroscience, Cognitive neuroscience

## Abstract

This study investigated if new dry-electrode technologies for electroencephalography (EEG) can substantially lower patient and site burden in clinical trials while maintaining adequate data quality. We benchmarked three dry-electrode EEG devices against a standard EEG using typical clinical trial procedures and EEG tasks that are often used for biomarker purposes. We found that dry-electrode EEG can perform on par with standard EEG for a range of different applications. However, both the participant and technician acceptance varied strongly across devices. Dry-electrode EEG was able to only match the comfort of standard EEG at best but was faster and easier to work with. Consequently, dry-electrode EEG was ranked among the most and least preferred options. The quantitative performance of dry-electrode EEG varied strongly across different applications. For example, quantitative resting state EEG and P300 evoked activity were adequately captured by dry-electrode EEG. However, certain signal aspects, such as low frequency activity (< 6 Hz) and induced gamma activity (40–80 Hz) presented notable challenges for dry-electrode EEG. Our findings suggest that dry-electrode EEG can substantially improve clinical trial applications of EEG, if the device and its context of use are carefully matched.

**Supplementary Information:**

The online version contains supplementary material available at 10.1038/s41598-025-18184-7.

## Introduction

The year 2024 marked the centenary of human Electroencephalography (EEG). After 100 years this non-invasive recording technique remains a mainstay for studying human neurophysiology and cognition^1^.

EEG provides direct access to human neuronal activity at millisecond resolution. Other key strengths are the comparably low cost and ease of access for multi-site studies, as well as the versatile use across age ranges (infants to elderly) and patient populations that can be assessed with electrode types and configurations matching the specific context. Together, these properties make EEG a powerful technique to support clinical trials. Examples for its use range from studying disease related phenotypes including sleep, to the pharmacodynamic effects of potential new therapies^2–13^.

In the context of clinical trials, minimizing patient and clinical site burden is key. Clinical site visits can be strenuous for patients who lead busy lifes and may suffer from severe conditions. Lengthy site visits, loaded with assessments may then cause inferior data quality. In some cases, patients may even be lost to follow up for a clinical trial.

Depending on the exact set-up and patient population, EEG can add burden to a clinical trial. Electrodes need to be placed carefully, often involving conductive paste and time consuming optimisation of recording conditions, followed by longer clean-up procedures for both patients and site staff.

The introduction of dry-electrode EEG systems may help to overcome some of these limitations, potentially offering improved ease and speed, as well as improved patient comfort^14–22^. Several studies report comparable signal qualities and usability of dry-electrode EEG as compared to standard EEG^15,16,18,22^. However, studies of dry-electrode EEG to date are mostly limited to research settings instead of clinical environments and often limited to particular hardware (also consumer grade) or study applications that may not translate well into a clinical trial setting.

The goal of this study is to provide a comprehensive, clinical trial-oriented comparison of state-of-the-art dry-electrode EEG devices against a standard wet EEG. Importantly, we sought to implement all procedures as closely to a clinical trial as possible, including data acquisition at a clinical testing site through staff participating in clinical trials. Instead of controlling for smaller technical differences between the devices, this study aims to describe the overall performance difference as they emerge according to the recommended use of the respective devices. In this way, the comparison between devices may offer an insight into the relative performance of the devices if a new study sponsor were to employ a particular solution. We focused the comparison on EEG recordings from tasks with relevance for biomarker purposes in early clinical trials (e.g. Phase 1 and 2), including resting state recordings as well as auditory and visually driven task related brain activity. We’d like to emphasize that the particular application of dry-electrode EEG for the study of specific patient populations (e.g. epilepsy, neurodegeneration, psychiatric disorders, pediatric indications, etc.) or purposes (sleep, acute monitoring, diagnostics, etc.) is beyond the scope of this study. This study may be representative of procedures in an early clinical trial in healthy volunteers, commonly performed to establish the safety and tolerability as well as the proof of mechanism for investigational drugs. As such, the resulting comparison between devices here may offer valuable insights for many studies also with different participant populations but the results cannot be fully representative of every potential application. Next to the EEG recordings, we also collected questionnaire data from participants and technicians to assess the relative burden and ease of use of the tested devices.

## Results

### Site and patient burden

**Fig. 1:**
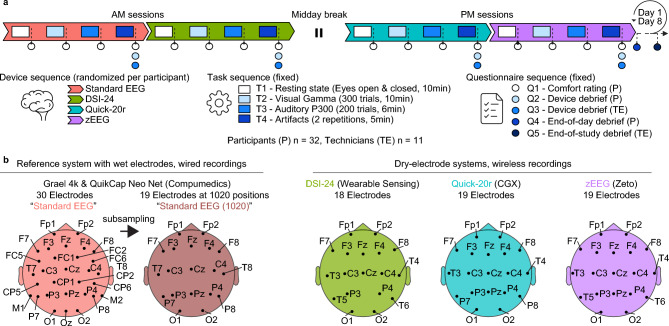
Study design and EEG devices tested in the study (**a**) Three dry-electrode devices and a standard EEG were included in the study. The color assignment is used consistently throughout all figures to refer to the different devices. Each of 32 participants was assigned a device order with a latin square randomization that was followed on both test (D1) and retest (D8) days. On each day both participants (P) and technicians (TE) filled in questionnaires and visual analog scale ratings (Q1-5) to indicate their perception of and opinions on the devices. On each recording day each participant was recorded with each device. EEG was recorded during four tasks in fixed order for each device (T1-4). (**b**) Electrode configurations for all devices tested in the study. The standard EEG was recorded with 19 electrodes at standard 1020 positions and 11 additional electrodes. Later analyses of EEG data are shown for both configurations separately to inspect the role of channel counts on data quality. The dry-electrode EEG devices all covered 1020 positions with differences in the exact channel availability. Please note that all channel labels and positions are based on manufacturer information and inconsistencies with the most current 1020 label definitions^23^ may exist.

To emulate clinical trials as closely as possible, all experiments were performed at a clinical testing site that is in routine use for early drug development (e.g. Phase 1 & 2 trials) by trained personnel, which is routinely involved in clinical trials and experienced with EEG. A total of n = 32 healthy participants completed two separate recording days with 3 dry-electrode (DSI-24, Wearable Sensing; Quick-20R, CGX; zEEG, Zeto) and 1 standard EEG device (QuikCap Neo Net with a Grael amplifier, Compumedics). The standard EEG served as a benchmark, allowing us to compare the new devices to typically employed procedures (Fig. 1).

**Fig. 2:**
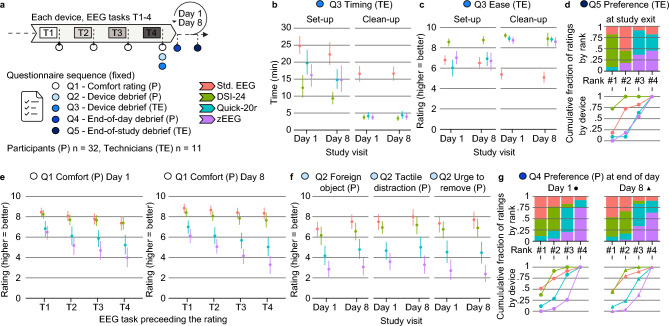
Questionnaire data from participants (P) and technicians (TE) as proxy for patient and site burden. (**a**) Sequence of questionnaires and ratings. For each device and after each task, participants made a comfort rating (see **e**). At the end of each device’s session, P and TE were debriefed with additional questions about their experience (see **b**,**c**,**f**). In addition, P were debriefed at the end of each recording day (see **g**) and TE were debriefed at the end of the study (see **d**). (**b**) Average set-up and clean-up times for each device across participants as measured by the technicians. Error bars reflect the 95% confidence interval. (**c**) Average technician ratings of the ease with which each device could be set-up and cleaned up. Error bars reflect the 95% confidence interval. (**d**) Technicians’ rank preferences across devices at the end of the study, where #1 is the most and #4 the least preferred device. The top panel shows the fraction of ratings for each device at each rank and the bottom panel shows the cumulative fraction of ratings across ranks for each device. (**e**) Average participants’ comfort ratings on both testing days. Comfort ratings were collected after each task, allowing for assessing the temporal evolution of comfort throughout the recording days. Error bars reflect the 95% confidence interval. (**f**) Average participant ratings on the degree to which a device felt like a foreign object, led to tactile distraction during the tasks or induced an urge to remove the device from the head. Error bars reflect the 95% confidence interval. (**g**) Participants’ rank preferences across devices at the end of each recording day, where #1 is the most and #4 the least preferred device. The top panels show the fraction of ratings for each device at each rank and the bottom panel shows the cumulative fraction of ratings across ranks for each device.

The burden of assessments is a key variable for the successful conduct of a clinical trial and can strongly influence the quality of clinical trial data. We explore the data from participants and technicians as a proxy for patient and site burden, respectively. We collected the opinions of participants and technicians about the EEG devices and information about operational aspects with structured questionnaires and rating scales (Fig. 2a).

We found that dry-electrode EEG sped up the experiments and was easier to work with for the technicians (Fig. 2b,c), in line with a high potential of dry-electrode EEG to substantially reduce the site burden during a clinical trial.

Set-up time was measured as the time elapsed from starting to prepare the EEG to starting the actual EEG recording. We found a significant impact of the devices on timing (χ^2^ = 70.56, df = 3, *p* < 0.001, Kruskal–Wallis test, pooled data across days), with all dry-devices being faster than the standard EEG (DSI-24 *p* < 0.001, Quick-20r *p* < 0.001, zEEG *p* = 1.15 × 10^–3^, Dunn’s tests, Holm adjusted). The fastest device required half the time as compared to the standard EEG (see Table 1) and was also significantly faster than the other dry devices (compared to Quick-20r *p* < 0.001, compared to zEEGp < 0.001, Dunn’s tests, Holm adjusted).

**Table 1:** Key questionnaire results pooled across study days.

Device	Set-up (technicians)		Clean-up (technicians)		Comfort (participants, rating 0–10)
	Time (min)	Ease (rating 0–10)	Time (min)	Ease (rating (0–10))	
Standard EEG	23.57 (21.46–25.81)	6.65 (6.28–7.03)	16.59 (14.99–18.21)	5.24 (4.87–5.65)	8.22 (8.02–8.41)
DSI-24	10.98 (9.19–13.46)	8.66 (8.38–8.93)	3.6 (3.05–4.21)	9.06 (8.66–9.35)	7.86 (7.66–8.06)
Quick-20r	17.42 (15.04–20.01)	6.44 (5.85–6.98)	4.24 (3.55–5.03)	8.86 (8.63–9.09)	5.98 (5.67–6.29)
zEEG	15.48 (13.04–17.98)	6.87 (6.32–7.43)	3.82 (3.17–4.46)	8.67 (8.37–8.96)	4.88 (4.54–5.18)

Data shown reflect the mean and the 95% confidence level.

Clean-up time was the time between the end of the recording and a fully cleaned set-up. We again found a significant impact of the devices on timing (χ^2^ = 133.91, df = 3, *p* < 0.001, Kruskal–Wallis test, pooled data across days). All dry-electrode devices achieved a comparable and substantial speed-up as compared to the standard EEG (see Table 1 DSI-24 *p* < 0.001, Quick-20r *p* < 0.001, zEEG *p* < 0.001, Dunn’s tests, Holm adjusted).

Importantly, the standard EEG had 11 additional electrodes as compared to the international 1020 montage that was covered by the dry-electrode devices (See supplementary information, study protocol, Sect. 7.2). The placement of these additional electrodes introduces an additional burden on timing for the standard EEG that needs to be considered when interpreting the above results. We chose the higher electrode count for the standard EEG, as it represents the most likely standard choice for clinical trials and the intended benchmark for comparisons. The higher channel count for the standard EEG allowed us to perform additional analyses on the EEG data, by synthetically reducing channel counts for the standard EEG to 1020 locations and to match the number of channels of the dry-electrode devices (Standard EEG (1020), Fig. 1b). Importantly, the subsampling was done before preprocessing the EEG data, such that the channel counts were equated to the dry-electrode EEG along the entire processing pipelines. For all EEG analyses below that could benefit from inspecting the impact of channel counts, we added the standard EEG (1020) next to the full standard EEG montage.

In line with the timing results, dry-electrode devices were easier to work with for the technicians. We found a significant effect of devices on the ease of set-up as reported by technicians (χ^2^ = 54.6, df = 3, *p* < 0.001, Kruskal–Wallis test, pooled data across days). Interestingly, only one device (DSI-24) could significantly differentiate itself from all other devices for the ease of set-up and was the easiest to work with (Scale from 0 (worst) to 10 (best); DSI-24 median 9, standard EEG median 7 and *p* < 0.001, Quick-20r median 7 and *p* < 0.001, zEEG median 7 and *p* < 0.001, Dunn’s tests, Holm adjusted).

We also found a significant effect of devices on the ease of clean-up as reported by technicians (χ^2^ = 132.65, df = 3, *p* < 0.001, Kruskal–Wallis test, pooled data across days). All dry-electrode devices had a comparable ease of clean up and were significantly easier to clean than the standard EEG (Standard EEG median 5, DSI-24 median 9 and *p* < 0.001, Quick-20r median 9 and *p* < 0.001, zEEG median 9 and *p* < 0.001).

Technicians (n = 11, each performing all experiments of at least 2 and at most 5 participants) ranked dry-electrode devices at both, most preferred and least preferred positions at the end of the study (Fig. 2d). These findings may be indicative of a large variability in user acceptance that is driven by other factors than speed or ease of use, such as perceived familiarity or device design features. A comprehensive assessment of the nature of such potential additional factors is beyond the scope of this study.

We next turned towards the participants’ perspectives as a proxy for patient burden in clinical trials. Standard EEG emerged as the overall most comfortable device for EEG assessments, that could at best be matched by dry-electrode EEG.

On both testing days and for each device, participants indicated their perception of the devices’ comfort with rating after each task (Fig. 2e). This allowed us to examine the temporal evolution of comfort during the recordings. We observed a declining trend in comfort over time.

Overall, the devices had a significant impact on perceived comfort during the recordings (χ^2^ = 297.21, df = 3, *p* < 0.001, Kruskal–Wallis test, data pooled across days and tasks). The standard EEG was significantly more comfortable than the dry-electrode devices (Scale from 0 (worst) to 10 (best); Standard EEG median 9, DSI-24 median 8 and *p* = 0.03, Quick-20r median 6 and *p* < 0.001, zEEG median 5 and *p* < 0.001, Dunn’s tests, Holm adjusted). The DSI-24 device was significantly more comfortable than the other dry-electrode devices (Quick-20r *p* < 0.001, zEEG *p* < 0.001^9^, Dunn’s tests, Holm adjusted) and the Quick-20r device was significantly more comfortable than the zEEG device (*p* < 0.001, Dunn’s test, Holm adjusted).

We found a comparable pattern of results when patients were asked how much the devices felt like a foreign object, led to tactile distractions during the EEG tasks or induced an urge to remove the device from the head (Fig. 2f) at the end of the testing days. The devices had a significant impact on the ratings (Foreign object χ^2^ = 76.2, df = 3, *p* < 0.001, Tactile disturbance χ^2^ = 85.19, df = 3, *p* < 0.001, Urge to remove χ^2^ = 84.15, df = 3, *p* < 0.001, Kruskal–Wallis tests, pooled data across days).

In line with the participants’ comfort ratings, the standard EEG and DSI-24 emerged as the most preferred devices on both testing days, followed by Quick-20r and zEEG (Fig. 2g).

In sum, dry-electrode EEG may lead to substantial improvements in site burden through gains in speed and ease. However, the devices tested here showed marked differences in the comfort for study participants, being able to at best match standard EEG.

### Resting state EEG

**Fig. 3:**
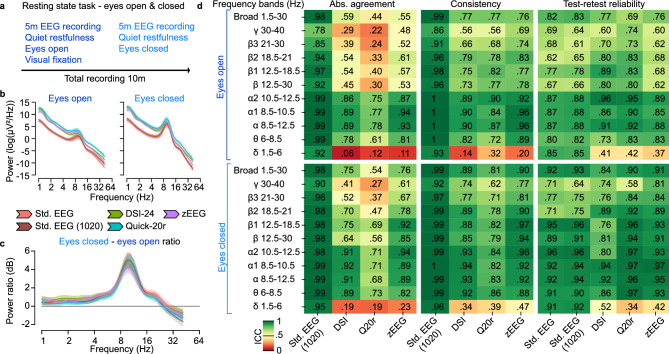
Resting state EEG. (**a**) Resting state EEG activity was recorded for 5 min during an eyes open and eyes closed condition. The participants fixated their gaze on a centrally displayed fixation dot during the eyes open condition. (**b**) Average power spectra during the eyes open and eyes closed conditions. Error bars reflect bootstrap estimates of the 95% confidence interval for the average across participants. Data was pooled across days and averaged across electrodes. (**c**) Average ratio of eyes closed to eyes open power. Error bars reflect bootstrap estimates of the 95% confidence interval for the average across participants. Data was pooled across days and averaged across electrodes. (**d**) Intraclass correlations (ICC) for power features derived for eyes open and eyes closed conditions. The absolute agreement and consistency are shown for each device as compared to the standard EEG. The test–retest reliability compared the features between the test (Day 1) and retest (Day 8) recordings. The standard EEG was also subsampled at only 1020 positions making the coverage comparable in number and locations to the dry-electrode EEG devices, see Std. EEG (1020).

We next turned towards the quantitative performance of dry-electrode EEG compared to standard EEG for assessing spectral power during eyes open and eyes closed resting state conditions (Fig. 3a).

The standard EEG included additional electrodes to the international 1020 Jasper montage (See supplementary information, study protocol, Sect. 7.2). In order to be able to assess the impact of only measuring signals at 1020 electrode locations as per dry-electrode EEG devices, we added analyses of the standard EEG limited to the 19 electrodes of the 1020 system (Standard EEG (1020), see Fig. 1b). Please note that no interpolation was performed for the standard EEG (1020) because only electrodes from the international 1020 Jasper montage were retained. The standard EEG (1020) was preprocessed separately from the standard EEG to allow for a full comparison between the montage with additional electrodes and the electrodes limited to 1020 positions. The comparison between the full standard EEG montage and the subsampled montage at 1020 electrode locations allows for assessing the impact on the quantitative results that go back to the channel count. We have added the standard EEG (1020) analyses for all subsequent EEG analyses where the channel count may contribute to the results.

All EEG devices measured the typical 1/f EEG power spectrum with a prominent peak in the 10 Hz alpha frequency range (Fig. 3b). Notably, all dry-electrode EEG power spectra had a consistent, positive broadband offset as compared to the standard EEG, likely reflecting the differences in sensor physics.

To assess the utility of the devices for measuring physiological modulations of brain activity, we computed the ratio between eyes closed and eyes open conditions (Fig. 3c). This contrast between conditions reveals the Berger effect—an increase in alpha frequency (~ 10 Hz) signal power upon closing the eyes. We found comparable signal modulations between the two experimental conditions for all devices.

Key for reproducible biomarker measurements in clinical trials is the comparability between measurements when made with different devices or when made at separate timepoints. As all participants were recorded with all devices on two separate days, we compared the quantitative results between the devices and each devices’ test–retest reliability (Fig. 3d).

We computed intraclass correlations (ICC) and assessed the absolute agreement and the consistency between the standard EEG and each of the dry-electrode EEG devices. As per convention^22^, we refer to ICC values below 0.5 as poor, between 0.5 and 0.75 as moderate, between 0.75 and 0.9 and good and above 0.9 as excellent.

The absolute agreement assesses to what degree the data is in an identical space, i.e. without any offsets with respect to each other and a slope of 1. In contrast, consistency assesses to what degree the measurements are in an equivalent space, i.e. the data may be offset between devices and have a different slope. In this way consistency is similar to a conventional Pearson correlation, whereas the agreement requires identity of the data. We used the absolute agreement between the recording days as a measure for test–retest reliability.

We found good to excellent agreement and consistency between the dry-electrode EEG and the standard EEG for frequencies in the theta and alpha activity ranges (6–12.5 Hz). In line with the broadband offset of the power spectra for dry-electrode EEG (Fig. 3b), we found mostly poor and occasional moderate absolute agreement between the dry-electrode EEG and the standard EEG for higher (> 12.5 Hz) and lower (< 6 Hz) activity. The absolute agreement tended to be generally higher during the eyes-closed condition, which may either be explained through additional physiological signals, reduced noise (e.g. movements, blinks) or a mixture of both. The consistency between dry-electrode EEG and standard EEG for higher frequency activity (> 12.5 Hz) was better than the agreement and mostly good. However, the consistency remained poor for lower frequency activity (< 6 Hz), suggesting the presence of idiosyncratic noise and non-replicable signal sources for the dry-electrode EEG. In line with these observations, the test–retest reliability also remained poor for the dry-electrode EEG for low frequency activity (< 6 Hz), while it was on par with the standard EEG and comparable for all devices for any higher frequency activity.

To study the impact of different noise sources on the EEG recordings, we included a task during which participants were asked to perform instructed movements that are known noise sources for EEG (See supplementary information, Fig S2-5). The results were consistent with a larger impact of movement related artifacts on dry-electrode EEG as compared to standard EEG, in particular for jaw clenching and full head movements.

In sum, dry-electrode EEG largely generates consistent and reliable resting state EEG features and may deliver equivalent biomarker results for a clinical trial in many applications. However, specifically for low frequency activity (< 6 Hz) the consistency and reliability are poor, which needs to be taken into account when considering whether dry-electrode EEG is fit-for-purpose for a specific clinical trial context.

### P300 auditory evoked potentials

**Fig. 4:**
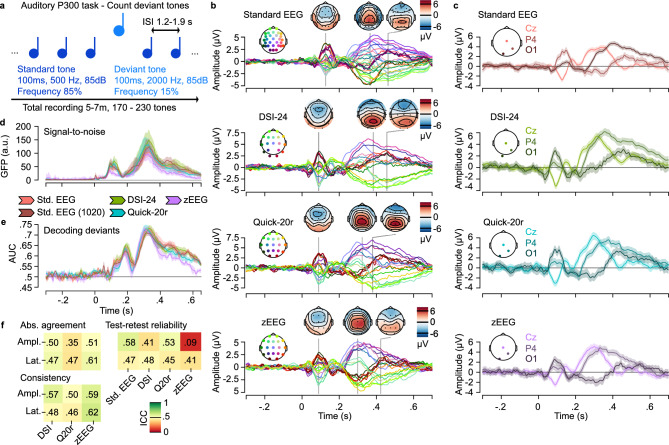
P300 auditory evoked potentials. (**a**) During the P300 task participants were asked to count deviant tones in a stream of standard tones. Overall, 25–35 deviant tones were recorded per participant and session. ISI is the interstimulus interval and was set to a random number between 1.2 and 1.9 s (**b**) Butterfly plots showing average voltage traces for each electrode per device as well as voltage topographies at select time points. All data shown is for deviant tones only. No error ranges for the averages over participants and sessions are shown. (**c**) Same data as in B shown for three electrodes that were consistently present across the different devices. Error bars reflect bootstrap estimates of the 95% confidence interval. (**d**) The global field potential as a measure of SNR for deviant tones per device. Error bars reflect bootstrap estimates of the 95% confidence interval. The standard EEG was also subsampled at the electrodes present consistently for the dry electrode devices, see Std. EEG (1020). (**e**) The area under the curve (AUC) for a logistic regression decoder, trained to identify deviant tones from standard tones based on the EEG topographies. Error bars reflect bootstrap estimates of the 95% confidence interval, same colors as in D. (**f**) Intraclass correlations (ICC) for latency (Lat.) and amplitude (Ampl.) features derived from the deviant evoked potentials. The features were derived as the time point and voltage amplitude of the maximal deflection within the 250–500 ms post stimulus onset period at the Cz electrode^24^. The absolute agreement and consistency are shown for each device as compared to the standard EEG. The test–retest reliability compared the features between the test (Day 1) and retest (Day 8) recordings.

We found comparable quantitative performance of dry-electrode EEG and standard EEG for recording evoked potentials during an auditory oddball paradigm^24^. Participants were asked to count deviant tones in a stream of standard tones occurring at random intervals while we recorded EEG (Fig. 4a).

All devices could recover well-known scalp-wide dynamics during the deviant tones with prominent P300 activity. Differences in the exact channel counts and positions led to nuanced differences in the overall morphology of the butterfly plots (Fig. 4b). When focusing on a subset of electrodes present across all devices, we observed overall highly comparable dynamics (Fig. 4c), suggesting a comparable utility of dry-electrode EEG as compared to standard EEG for biomarker purposes based on evoked potentials.

We investigated the global field potential (GFP) and the decoding performance of a logistic regression (decoding deviants from standards) as additional metrics for comparison (Fig. 4d,e, see Methods, Statistical analyses for details). The GFP offers a direct metricof SNR of the measurements,comparing variances between baseline and stimulus period^25^. Briefly, the GFP quantifies deviations of the variability across the sensors of a device as compared to a reference (baseline) period and, thus, is a suitable metric to monitor global signal across an EEG montage for evoked potentials. l Complementing the GFP, the decoding performance of a logistic regression offers a direct quantification of the amount of information (i.e. discriminatory power) that can be extracted from the different devices about physiologically and clinically meaningful task related activity.

We found that the average GFP in a 100 ms time window around the P300 peak differed between devices (Fig. 4d) with the zEEG showing the lowest SNR and the DSI-24 showing the highest SNR (χ^2^ = 281.4, df = 4, *p* < 0.001, Kruskal–Wallis test, see Table 2) These differences persisted within a later time window from 300–600 ms post-stimulus (χ^2^ = 372.89, df = 4, *p* < 0.001, Kruskal–Wallis test) ;. Of note, the standard EEG and subsampled standard EEG (1020) exhibited strong differences in GFP that may relate to the differences in channel counts. The GFP computation involves a normalization with channel counts (see Methods, Statistical Analyses). Plausibly, the subsampled standard EEG (1020) captures all relevant EEG signal, yet contributes a lower channel count to the normalization. A lower ratio between electrodes that do and do not have task related brain activity for the standard EEG will then explain the differences in GFP that result from differences in channel counts exclusively. For this reason, the standard EEG (1020) with equated channel counts to all other devices represents the relevant reference for comparisons of the GFP metric between standard EEG and dry-electrode EEG. Please also note that the montage of the DSI-24 contained 18 electrodes (lacking Pz) while the other devices contained 19 electrodes (Fig. 1b). This 1 channel difference may have contributed a boost in GFP magnitude for the DSI-24 along the same reasoning as above. However, based on the magnitude of the GFP differences for the 11 electrode discrepancy between the standard EEG and the standard EEG (1020) a 1 electrode difference may only contribute a minor effect.

**Table 2:** SNR as captured by the GFP and amounts of stimulus information during peak and later trial periods.

Device	100 ms around P300 peak		300–600 ms post stimulus	
	GFP (95% CI)	AUC (95% CI)	GFP (95% CI)	AUC (95% CI)
Standard EEG	86.26 (84.39–88.18)	0.70 (0.68–0.72)	53.82 (52.95–54.70)	0.66 (0.64–0.67)
Std. EEG (1020)	98.92 (96.39–101.40)	0.68 (0.67–0.70)	67.63 (66.25–69.01)	0.65 (0.64–0.66)
DSI-24	118.39 (114–122.29)	0.69 (0.68–0.7)	71.54 (70.02–73.29)	0.65 (0.64–0.66)
Quick-20r	108.2 (105.42–110.84)	0.7 (0.68–0.72)	57.66 (56.16–59.08)	0.63 (0.62–0.65)
zEEG	81.23 (79.13–83.33)	0.69 (0.68–0.7)	67.36 (65.99–68.78)	0.61 (0.6–0.62)

The amount of information in a 100 ms time window around the P300 peak was comparable for all devices (χ^2^ = 2.87, df = 4, *p* = 0.58, Kruskal–Wallis test). Differences in task related information emerged within the later time window from 300–600 ms (χ^2^ = 12.056, df = 4, *p* = 0.017, Kruskal–Wallis test , , with the zEEG device showing a more substantial loss of information at this later time in the trial (See Table 2). Interestingly, subsampling the standard EEG at 1020 locations (standard EEG (1020)) did not lead to any measurable loss in information (Fig. 4e), suggesting that the channel counts of the dry-electrode EEG devices was in principle sufficient to recover all relevant task information during the auditory oddball paradigm. This analysis is complementary to the previous comparison of GFP suggesting that despite discrepancies in SNR, all devices measured a similar amount of task specific information.

Lastly, we studied the agreement, consistency and test–retest reliability of the P300 evoked potential maximum amplitude and latency in the 250-500 ms post stimulus period^24^—two features with potential relevance as biomarkers for clinical trials^26^ (Fig. 4f).

Table 3 summarizes the extracted features for each device. The P300 latencies were comparable between all devices (χ^2^ = 2.88, df = 3, *p* = 0.41, Kruskal–Wallis test, see Table 3).

**Table 3:** Average latency and amplitude features for the P300 per device.

Device	P300 latency (95% CI)	P300 amplitude (95% CI)
Standard EEG	0.31 s (0.30 s–0.32 s)	5.46 μV (4.88 μV–6.28 μV)
DSI-24	0.32 s (0.31 s–0.33 s)	6.81 μV (6.16 μV–7.50 μV)
Quick-20r	0.31 s (0.30 s–0.33 s)	8.01 μV (7.13 μV–8.91 μV)
zEEG	0.32 s (0.30 s–0.33 s)	6.54 μV (5.88 μV–7.26 μV)

The devices had a significant impact on the measured P300 amplitudes at maximum (χ^2^ = 21.75, df = 3, *p* < 0.001, Kruskal–Wallis test see Table 3) with the Quick-20r measuring the largest and the standard EEG measuring the smallest P300 amplitudes.

The intraclass correlations were generally low and mostly moderate to poor across all devices, including the standard EEG (Fig. 4f). Among mostly comparably low intraclass correlation values, we observed one considerably lower value for the test–retest reliability of the latency for the zEEG device.

Next to inter and intra-individual variability, a key factor for the low reliabilities may be the relatively low number of deviant tones per participant and session in this study (n = 25 to 35). The intraclass correlations may emerge to be more robust when additional trials are contributing to the analyses and were previously found to be able to achieve good-to-excellent reliabilities^27^.

In sum, all devices showed comparable magnitudes of information for the auditory oddball paradigm. These findings suggest a mostly equivalent utility of dry-electrode EEG as compared to the standard EEG for the auditory oddball task as employed here. Improved robustness of the evoked potential features latency and amplitude during a clinical trial may require the acquisition of more data per participant than was collected here.

### Visually driven EEG activity changes

**Fig. 5:**
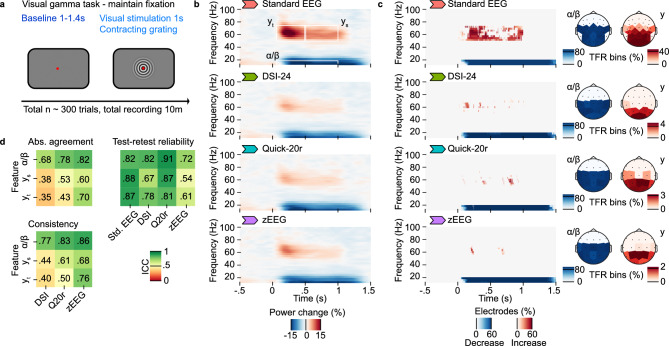
Visual gamma activity. (**a**) Participants quietly maintained fixation on a centrally displayed fixation dot without additional active behavioral tasks. After a variable baseline, a visual stimulus consisting of a contracting grating was displayed around the fixation dot for 1 s. (**b**) Time frequency representation (TFR) of EEG activity averaged across all available trials and recordings for electrodes that are part of a posterior-occipital region of interest available across devices (102,010–20 system electrodes O1, O2, P3, Pz, P4, C3, Cz, C4). White boxes define the definitions of the TFR features used for ICC analysis (see **d**), where the same electrodes and the respective time frequency bins within the indicated area of the TFR have been averaged per patient. Combined alpha and beta activity (ɑ/ꞵ) was defined as 0.05–1.5 s post stimulus onset and 10–20 Hz. Transient gamma activity (γ_t_) was defined as 0.05–0.5 s post stimulus onset and 50–75 Hz. Sustained gamma activity (γ_s_) was defined as 0.5–1 s post stimulus onset and 50–75 Hz. (**c**) Robustness and extent of the induced activity per device. Results from a non-parametric cluster based significance test of the signal power change across the entire time, frequency and electrode space. For all significant clusters (p_FWER_ < 0.05, random permutation test), percentages of contributing electrodes (for the TFR) and time–frequency bins of the TFR regions of interest (for the topographies) are being displayed. The TFR regions of interest are defined in b. Clusters with a positive change from baseline are depicted in red color, clusters with a negative change from baseline are depicted with a blue color. (**d**) Intraclass correlations (ICC) for alpha (ɑ), transient (γ_t_) and sustained gamma (γ_s_) power change (see B). The absolute agreement and consistency are shown for each device as compared to the standard EEG. The test–retest reliability compared the features between the test (Day 1) and retest (Day 8) recordings. The standard EEG was also subsampled at the electrodes present consistently for the dry electrode devices, see [Std. EEG (1020)], to allow for a comparison of ICC values based on comparable electrode configurations between devices.

We next investigated the quantitative performances during a visual task (Fig. 5a). We used contracting circular gratings as visual stimuli to induce a canonical pattern of EEG activity changes in the alpha (10 Hz), beta (20 Hz) and gamma (60 Hz) frequency ranges^28,29^. Detecting visual gamma activity changes in EEG is a challenging task^30^, and may be a discriminative benchmark of different EEG devices.

All devices qualitatively showed the pattern of visually driven EEG activity changes with increased gamma activity (around 60 Hz) and decreased alpha/beta activity (10–20 Hz) for an occipital group of channels (Fig. 5b). However, the magnitudes of change, in particular in the gamma range differed across devices, with dry-electrode EEG generally showing lower magnitudes of change from baseline than the standard EEG.

In order to assess the robustness of the activity changes in a data driven way, we used cluster-based permutation statistics (Fig. 5c). All devices were able to significantly quantify both increased gamma and decreased alpha/beta activity changes (p_FWER_ < 0.05, random permutation tests). However, the overall extent of significant changes in gamma activity were much smaller and constrained to brief periods of the visual stimulation for the dry-electrode EEG devices as compared to the standard EEG.

As for the other tasks, we next assessed the agreement and consistency between dry-electrode and standard EEG as well as the test–retest reliability of the gamma and alpha/beta activity for all devices (Fig. 5d).

For the alpha/beta activity changes we observed mostly good agreement and consistency between dry-electrode EEG devices and the standard EEG. A pattern of increasing intraclass correlation values emerged that placed the DSI-24 at the lowest scores, Quick-20r at intermediate scores and the zEEG at the highest scores of agreement with the standard EEG. This pattern was even more pronounced for the gamma activity where the consistency with the standard EEG dropped to poor while it remained at moderate to good levels for the zEEG.

Interestingly, this overall pattern across devices was in reverse order as compared to the perceived comfort of wearing the devices by participants (Fig. 2E,F). These observations may indicate a more general trade-off between patient burden through the device and data quality for detecting more subtle brain activity changes across different dry-electrode EEG solutions.

The test–retest reliability ranged from moderate to excellent with the standard EEG and Quick-20r scoring the highest reliability followed by DSI-24 and the zEEG with the lowest scores.

In sum, dry-electrode EEG may show good utility for assessing visually induced alpha/beta activity changes with close to equivalent quantitative performance as compared to the standard EEG. However, the ability to quantify gamma activity is strongly diminished for dry-electrode EEG, suggesting that the use of dry-electrode EEG needs to be matched to the visually induced brain activity changes of interest.

## Discussion

We benchmarked the performance of three dry-electrode EEG devices against a standard EEG across three clinically relevant tasks, using procedures typically applied in early clinical trials. We found that dry-electrode EEG could occasionally deliver on par performance as compared to the standard EEG. Substantial differences in terms of comfort and quantitative performance emerged that were highly dependent on the specific device and task. Our results suggest that dry-electrode EEG can lead to substantial improvements in EEG assessments for clinical trials, if the specific device and its context of use are considered carefully.

We found that dry-electrode EEG devices were easier and faster (factor of 2–fivefold) to work with, consistent with previous assessments in research grade settings^15,16^. These properties may be the key operational advantages over standard EEG and reduce patient and site burden during clinical trials. Overall, these advantages may potentially outweigh differences in costs for the instrumentation, a determination that will require a case-by-case comparison of wet vs. dry EEG options. Interestingly, the dry-electrode EEG devices could at best match the standard EEG in terms of comfort during the recordings, with substantial differences between the dry-electrode EEG devices. Consequently, dry-electrode devices ranked both as the most as well as least preferred device for technicians and about half of the study participants liked the standard EEG best. The discomfort with dry-electrode solutions may, however, not play an equal role during all EEG applications. For example, tactile distraction and urges to remove the devices from the head may confound assessments of subtle cognitive effects more strongly than pharmacodynamic measurements where strong drug-related effects can be expected. At the same time, longer setup for wet EEG could potentially also improve patient rapport as contact and the perception of closer care may be considered positive aspects of the assessment. In that way, short set-up time and discomfort could be more critical in clinical populations, which should be considered on a case-by-case basis.

Another important factor for considering the questionnaire data of this study is prior experiences with EEG. All technicians were trained and experienced with standard EEG procedures. As a result, their preferences may have partially been affected by their previous professional experiences. Such effects of previous experiences are more unlikely for study participants who were mostly naive to EEG altogether. Generally, the prior experience by technicians and participants in this study may be comparable to that in other studies that aim to implement dry-electrode recordings in the future. In this way, the key results from the questionnaire data here may potentially generalize to future studies.

Overall, the findings suggest that the choice of a dry-electrode device needs to consider how the hardware may impact the study conduct in ways that are not related to the speed of set-up and clean-up or its general ease of use.

All dry-electrode devices were able to measure resting state brain activity in clinically meaningful frequency ranges with good-to-excellent consistency and reliability. The standard EEG showed a stronger resilience to noise sources and largely outperformed the dry-electrode EEG devices for higher and low frequency activity. These differences may be related to a more reliable coupling between sensor and scalp for the standard EEG (conductive paste) and reduced susceptibility to head movement. The susceptibility to artifacts and head movements for dry-electrode devices became particularly apparent during the artifact task where dry-electrode EEG showed strong broadband differences as compared to standard EEG in particular for the head-movement and jaw-movement conditions. These findings again suggest that the choice of dry-electrode EEG for resting state recordings needs to consider the exact context of use. Low frequency activity changes that can constitute a central phenotype of neurodegenerative^31^ and neurodevelopmental diseases^8^ may be difficult to study with current dry-electrode EEG systems. At the same time, if the physiological signature of interest involves theta to alpha activity^7^, dry-electrode EEG may be an attractive alternative to standard EEG.

We found that evoked activity in a P300 auditory oddball paradigm was equivalently captured across devices, with all devices showing evoked potentials with mostly comparable key features and amounts of task related information in this particular task implementation. While the amount of information extracted from a logistic regression decoder was comparable across devices, the specific amplitudes for the P300 differed across devices along the SNR as assessed through the global field potential (GFP). These signal differences may potentially have resulted from differences in synchronization and internal device gains between the devices. Interestingly, the P300 evoked activity that largely consists of lower frequency activity was captured similarly well across the devices despite the poor agreement and test–retest reliability for low frequency activity (< 6 Hz) during the resting state task (Fig. 3d). An important difference for the P300 task may be the larger task evoked brain activity occurring reliably and consistently in time in addition to the averaging across trials. These factors may contribute a signal boost for the dry-electrode devices making them perform adequately for assessing the P300. However, the consistency and reliability of the P300 measures also need to be taken into account.

The consistency and reliability of the evoked potential features was at best moderate for this task for all devices, potentially linked to a relatively low number of trials recorded per participant. The low trial count was sufficient for device comparison purposes presented here, but needs to be higher if evoked activity is studied as a trial endpoint. Our data suggest that the choice of a particular dry-electrode device may not critically influence the recording outcomes of auditory evoked potentials and that dry-electrode devices may perform on par with standard EEG for this application.

We used a visual paradigm to induce occipital gamma activity, which represents a challenging signature to detect in EEG recordings^28,32^. While gamma activity was generally detectable with all devices, only the standard EEG captured a sustained and robust gamma response during the visual stimulation period. All devices captured induced changes in the alpha and beta frequency ranges with good agreement and reliability. Thus, in line with the previous findings, the choice for dry-electrode EEG needs to consider the specific context of use, with gamma activity being difficult to detect, while alpha and beta frequency changes being detectable in an equivalent way by dry-electrode and standard EEG.

It is important to note that the results of this study come with limitations. All results reflect data obtained in healthy volunteers and in a mainly caucasian population. The application of dry-electrode EEG to particular patient populations in neurology or psychiatry may require additional considerations that were beyond the scope of this study. In addition, how hair styles and hair thickness contribute to differential EEG device performances may play an important role when studies are planned globally. Furthermore, this study investigated only a limited number of endpoints (tasks) with specific choices for their implementation, such as interstimulus intervals and other parameters. Thus, the generalizability of our findings to other endpoints or implementations may be limited and we recommend a careful consideration of the intended use of dry-electrode EEG with regard to the patient population and endpoints for a new study.

A source of potential biases in this study may also stem from the automated preprocessing pipeline, that has used a random forest (RF) model trained on a wet electrode EEG system that has not been used as a part of this study. While we do believe that the preprocessing has not introduced undue disadvantages for the comparison between dry-electrode devices, the training on wet EEG data may lead to favorable processing for more similar standard wet EEG devices. Similarly, the pre-existing training and experience of technicians with standard wet EEG may bias their perception towards similar devices and should be taken into account when interpreting the differences between standard EEG and the dry-electrode EEG devices.

In sum, our findings show a high potential for dry-electrode EEG to substantially improve clinical trial applications of EEG. However, current systems can at best match the user acceptance and quantitative performance of standard EEG. The use of dry-electrode EEG needs to be carefully matched to the context of use and scientific question at hand with both the patient population as well as EEG derived biomarker signal in mind. We hope that the procedures laid out here can contribute to informing further improvements in novel EEG sensor technology that support the use of this rich and versatile human neuroimaging technique in clinical trials. In the future, a broader use of EEG for clinical monitoring outside of clinical sites with specialty in EEG as well as at-home recording through patients for more ecologically valid data may be important areas. In parallel to these developments, cost-conscious hardware solutions and robust analysis tools are required to help clinical sites manage the cost and burden of these new technologies.

## Methods

### Study design

All experiments were conducted through Biotrial at a single clinical trial testing site in Rennes, France. Biotrial is an experienced Contract Research Organization (CRO) for clinical trials in neuroscience with experience in the use of EEG to assess pharmacodynamics and biomarkers during drug development. The use of a CRO and clinical trial testing site were meant to enable the resulting data to be as comparable to clinical trial data as possible and enable an evaluation of the EEG devices for this setting.

We obtained approval from the ethics committee (Comité de Protection des Personnes Nord Ouest IX, Lille, France) prior to study start as well as informed consent from all study participants. All experiments were conducted according to the Declaration of Helsinki and adhered to relevant guidelines and regulations. The full study protocol is available as supplementary information (See supplementary information, study protocol) to enable the replication of all findings.

We conducted a landscaping search for EEG devices and performed pilot testing prior to the full study in order to support the final selection of devices into the study. The criteria for device selection were 1) a clear innovative advance in data acquisition technology, such as the use of dry-electrode sensors and wireless recording options and 2) general adherence to the international 1020 Jasper montage for comparability to standard practice. Additional factors for device selection included the potential readiness and operational feasibility for clinical trial support, including health authority certification such as available FDA 510(k) clearance and CE marking. Together these criteria focussed the device selection on research oriented technology and excluded consumer-grade devices. More details on the selected devices are available in the supplementary information (See supplementary information, study protocol, Sect. 7). Briefly, the included dry-electrode EEG devices were the DSI-24 (Wearable Sensing, CA, USA), Quick-20r (CGX, CA, USA) and the zEEG (Zeto, CA, USA), with the Quik Cap and Grael amplifier (Compumedics, NC, USA) as the state-of-the-art standard EEG reference used by the clinical study site. Importantly, rather than trying to compare devices with an exactly equated hardware set-up (e.g. channel counts) at a detailed technical level, we here intended to use each device according to its own design and optimal set-up such that we could compare the overall clinical utility that resulted when employing the different devices. Please note that the devices differ in several hardware and data acquisition aspects from each other. For example, the channel count of the standard EEG was higher (32 electrodes) than those for the dry-electrode EEG devices. Please also note, that all technicians in the study (n = 11) were trained and had experience with the use of the standard EEG system in this study. All technicians also underwent a familiarization training with the devices and their specific hardware set-up ahead of the start of the study.

A total of 39 participants were screened for inclusion into the study. 35 participants (18 females, age range 19–45) successfully completed the first recording day and three participants (1 female) were lost to follow up after the first recording day, resulting in a total of 32 participants having completed both recording days of the study. We included healthy volunteers, giving informed consent, aged 18–45 years inclusive with a head circumference between 54 to 60 cm, who were able to undergo the study assessments. Exclusion criteria included hairstyles incompatible with EEG recordings, a history of neurological or psychiatric disorders and CNS active medication, participation in an investigational drug or device study within the prior 4 weeks (or 5 times the drug half-life). We requested study participants to abstain from the consumption of alcohol, caffeine and xanthine-containing products within 48 h prior to each recording visit and excluded participants in case of a positive test for drugs of abuse or alcohol. Please see the study protocol for additional details (See supplementary information, study protocol, Sect. 6).

### Study tasks

Additional details on all study procedures, questionnaires and tasks are included in the respective results sections as well as the study protocol (See Fig. 1, supplementary information, study protocol, Sect. 10.4).

We included four different tasks while EEG recordings were performed: A resting state task (Fig. 3a), an auditory P300 task (Fig. 4a), a visual task for inducing gamma activity (Fig. 5a) as well as an additional artifact task (Fig. S2).

The resting state task required participants to remain quietly awake while EEG was recorded. We performed 5 min of eyes closed and eyes open resting state recordings. During the eyes open condition participants were asked to fixate their view on a visually presented target.

For the P300 task participants were equipped with headphones and were instructed to close their eyes and remain still. Two types of 100 ms duration tones (500 Hz, 2000 Hz) were presented in a serial stream with a 1.2 s–1.9 s interstimulus interval at a sound pressure level of 85 dB. The standard tone (500 Hz) occured more commonly and a number of deviant tones between 25–35 was randomly selected and adjusted to represent a stimulus frequency of approx. 15%, leading to a total duration time that varied between 5 to 7 min. Participants were asked to count the deviant tones.

The visual task included annular, concentric, contracting gratings at a high contrast, known to robustly elicit gamma activity in occipital regions^28^. The gratings were shown on a monitor placed at a distance of 69 cm from the participants and with a size of 5 degrees visual angle. The spatial frequency was 2 cycles per degree and the movement speed was set to 3 cycles per second with a contrast of 100%. Participants kept their gaze fixated on a red fixation dot while each stimulus was presented for 1 s. Overall, 300 stimuli were presented with an interstimulus interval of 1 s–1.4 s.

For the artifact task EEG was recorded while participants went through five task conditions, prompting them to exhibit different degrees of movement types, commonly known to induce artifacts in the EEG. As a low-noise reference recording, the sequence of task conditions began with an eyes open resting state condition according to the resting state task procedures (see. Figure 2). The resting state recording was followed by visually instructed blinks and horizontal as well as vertical saccades, jaw clenching and lastly large whole head movements. The head movements included three different conditions with rotational movements around the yaw axis (a ‘No’ gesture), rotational movements around the pitch axis (a ‘Yes’ gesture) and forth and back movements with the entire upper torso, including the head (‘Bowing’). The sequence of task conditions was repeated once, leading to two recordings per condition.

### Signal processing

We developed an automatic preprocessing pipeline for cleaning the EEG data (See supplementary information, Fig. S1, Section S1) using openly available software (see below) as well as custom code.

For all tasks, preprocessing included bandpass filtering (1–45 Hz for resting state, 1-60 Hz for P300, 1–120 Hz for visual gamma, Hamming windowed Finite Impulse Response filter^33^) and the rejection of bad EEG channels based on extreme amplitude values and signal power. Specifically, a channel was labeled as bad if more than 20% of two second epochs showed either variance below 0.1 (flat signal), maximum amplitudes > 500 mV or amplitudes > 100 mV while also showing a correlation below 0.6 with each other channel. After channel rejection we performed a flat segment rejection by measuring the variance in 200 ms sliding windows for each consecutive two second segment and each channel. If any window within the segment had a variance lower than 0.1 the segment was rejected as flat. We then performed an independent component analysis (ICA), followed by classification and removal of independent components using a neural network (ICLabel^34^), which recognizes ocular, muscular, and cardiac artifacts. We performed an additional bad segment rejection step, using a Random Forest classifier that was trained on manually annotated EEG segments. The data for training and testing the Random Forest classifier was composed of the EU-AIMS LEAP data set^35^ and the Phase 1 dataset for Alogabat. Both trials used gel-based EEG systems that are different from any device used in this study. It is important to note that theoretically, the automatic preprocessing may have introduced potential differences in the EEG data between devices due to biases in the Random Forest model. However, the model used robust features for the rejection and comparable biases may be present for purely manual, i.e. subjective, preprocessing (also see supplementary information, Section S1 for additional preprocessing results). The classifier utilized 14 features, including the joint probability of EEG and ICA time series, the kurtosis of EEG and ICA time series, the maximum and minimum amplitude of EEG and ICA time series, the mean deviation from channel means, epoch variance, and the maximum amplitude difference^36^. Finally, we imputed signals from bad channels using spherical interpolation and re-referenced to the average reference. It is important to note that while the interpolation is a standard preprocessing approach, it may in principle have introduced differences for devices with differing channel counts. In this way the interpolation could potentially contribute to any observed differences in the devices’ signals.

For analyses where we restricted the standard EEG to the electrodes at 1020 positions , the extra channels were removed before preprocessing. This ensured a fair comparison by preventing any preprocessing advantages (e.g., improved ICA decomposition) that could arise from a higher channel count.

We used Morlet wavelets as implemented in the openly available package MEEGLET^37^ [https://roche.github.io/neuro-meeglet/] for frequency transforms of the resting state EEG data. Power spectra were obtained using 45 wavelets with a logarithmic spacing in octaves between 1 and 45 Hz, corresponding to a bandwidth of 0.5 octaves. For the visual gamma task we used multitaper spectral analysis, in keeping with published procedures for this task^28^. Briefly, we used 400 ms time windows sliding in 50 ms steps and selected frequencies between 5 and 120 Hz in 2.5 Hz steps with a spectral smoothing of ± 5 Hz obtained with multi-tapering.

### Statistical analyses

We report 95% confidence intervals as errors for all summary statistics. We estimated all confidence intervals using bootstrapping through random sampling with replacement.

We quantified the agreement and consistency between devices as well as the test–retest reliability of each device using intra class correlation coefficients^38,39^. To compare measurements between the dry-electrode EEG devices and the standard EEG, we used a two-way mixed effects model with participants as random effect and devices as fixed effect. We report on the absolute agreement as well as the consistency for single measurements throughout all analyses. In order to assess the test–retest reliability of each device we also used a two-way mixed effects model, with participants as random effect and sessions (Day 1 and Day 8) as the fixed effect. We report the absolute agreement of single measurements as the assessment of test–retest reliability throughout all analyses.

Each task condition required the extraction of different features (endpoints) that may potentially be used as biomarker signals in clinical trials.

For the resting state EEG task we used signal power features obtained from Morlet wavelets and averaged according to IPEG guidelines^40^.

For the auditory P300 task we extracted the maximal amplitude and its latency at the electrode Cz in the 0.25–0.5 s post stimulus as features^24^. In addition we assessed the global field potential (GFP) that allows for a comparison of the signal-to-noise ratio (SNR) across different devices^25^. Please note that we corrected a known technical 33 ms latency related to the set-up for the standard EEG by shifting the standard EEG analyses accordingly.

The GFP quantifies the signal strength post-stimulus in comparison to the baseline period. To achieve this, we first estimated the empirical noise covariance matrix using the baseline period from − 0.3 to 0 s before the auditory stimulus. The noise covariance matrix captures the variability and correlation of the noise in the baseline period. We then derived a whitener from this covariance matrix, which is a transformation that normalizes the noise, making it have a standard normal distribution. This whitener is applied to the entire trial, extending up to 0.7 s post-stimulus. If the rest of the signal were to follow the same distribution as the segment used for covariance estimation, the whitened data should follow the same standard normal distribution. GFP is a measure of the overall activity in the brain, computed as the sum of the squared signal across all electrodes. In this context, the GFP of the whitened data constitutes a chi-square test under the null hypothesis that the post-stimulus data follows the same distribution as the baseline. This approach was initially proposed to measure the quality of covariance estimates and is repurposed here to quantify the SNR by measuring how much the evoked potential deviates from baseline activity.

$$\begin{array}{*{20}c} {C = \frac{1}{{T_{b} }}X_{b} X_{b}^{ \top } \;{\text{with}}\;X_{b} \in R^{{P \times T_{b} }} } & \hbox{Empirical\;Covariance} \\ \end{array}$$

EEG data from the baseline period and $C$ denotes the whitening matrix

$$\begin{array}{*{20}l} {X_{w} = C^{{ - \frac{1}{2}}} X} \hfill & \hbox{Whitening} \hfill \\ \end{array}$$

$$\begin{array}{*{20}c} {GFP = \frac{1}{P}trace \left( {X_{w } X_{w}^{ \top } } \right) } & \hbox{Global\;Field\;Potential} \\ \end{array}$$

$X \in R^{P \times T}$ EEG data recorded with $P$ sensors and $T$ time points.

In addition to the GFP analysis that focused on the SNR, we also quantified the amount of task relevant information from the EEG activity using a decoding analysis. We trained a decoder for predicting the stimulus type (standard or deviant tone) from the instantaneous potential recorded over all electrodes. We used a logistic regression classifier with L2 regularization at each sample along the trial and quantified the Area Under the Curve (AUC) of the receiver operating characteristic (ROC) as a measure of information content in the EEG data. The AUC has a value of 0.5 under the null-hypothesis of no predictive power and increases from 0.5 reflect non-chance amounts of information about the tone type in the EEG data. We used a five-fold cross-validation scheme for estimating the AUC values.

For the visual gamma task we derived the features as the average signal power measured in three different time–frequency bins. We refer to these bins as alpha/beta (10–20 Hz from 0.05 s to 1.5 s), transient gamma (50-75 Hz, from 0.05 s to 0.5 s) and sustained gamma (50-75 Hz, from 0.5 s to 1 s) broadly consistent with the canonical response in brain activity as measured with EEG during visual stimulation^28,29^. Since the brain response to visual stimulation is known to exhibit an occipital and parietal topography we averaged the time–frequency bins over a subset of 8 channels consistently present in all devices: O1, O2, P3, Pz, P4, C3, Cz, and C4.

We conducted a paired t-test to compare each time–frequency bin during visual stimulation against the average for each frequency over the baseline period (Fig. 5b). To identify statistically significant changes in brain activity during visual stimulation, we employed cluster-based permutation statistics^41^. This method captures significant activity changes regardless of their specific time or frequency localization. Clusters were defined as neighboring bins in the time, frequency, and sensor space, with a test statistic greater than a given threshold. The notion of neighborhood was based on both the sensor adjacency matrix and direct proximity in time and frequency. We set the cluster forming threshold to 1.28, corresponding to the 90th percentile of the standard normal distribution. Sensitivity analyses confirmed that this threshold had minimal impact on the overall analysis (data not shown). To determine the significance of the clusters, we computed the permutation distribution of the cluster-level statistics, specifically the maximum absolute value within each cluster, over 1000 permutations. Figure 5c illustrates the significant clusters in the time–frequency domain, with colors indicating the percentage of electrodes contributing to the binary representations at each time–frequency bin. Figure 5d shows the topography of these binary representations, with colors representing the percentage of time–frequency bins contributing at each electrode.

### Software

We used MATLAB (Version R2023b, The Mathworks, Natick, MA, USA, https://de.mathworks.com/products/matlab.html), R Studio (Version 4.3.3, Boston, MA, USA, https://posit.co/download/rstudio-desktop/) and Python (Version 3.11, https://www.python.org/) with custom code as well as software packages available online for the data analysis of this study (Table 4).

**Table 4:** Software packages used for data analysis.

Name	Key reference	Weblink
MNE python	^42^	https://mne.tools/stable/index.html
Pingouin	^39^	https://pingouin-stats.org
MEEGLET	^37^	https://roche.github.io/neuro-meeglet/
EEGlab	^33^	https://eeglab.org/
IClabel	^34^	https://labeling.ucsd.edu/tutorial
statsmodels	^43^	https://github.com/statsmodels/statsmodels/

## Supplementary Information

Supplementary Information 1.

Supplementary Information 2.

## Data Availability

The dataset generated and analysed during the current study is not publicly available due compliance with study participant privacy but is available from the corresponding author on reasonable request.
