# Supplementary material for: Benchmarking the utility of dry-electrode electroencephalography for clinical trials
Source: Sci Rep. 2025 Sep 29;15:33667. doi: 10.1038/s41598-025-18184-7 (PMC12480468; doi:10.1038/s41598-025-18184-7)
Supplement: Supplementary file 1 — Supplementary Information 1. [file 41598_2025_18184_MOESM1_ESM.pdf]

Supplementary information – Supplementary results

Benchmarking the utility of dry electrode electroencephalography for clinical trials

Joseph Paillard*[1], Philipp Bomatter*[1,2], Laura Dubreuil-Vall[1,3], Jörg F. Hipp[1], David J. Hawellek[1&]

Automated EEG preprocessing

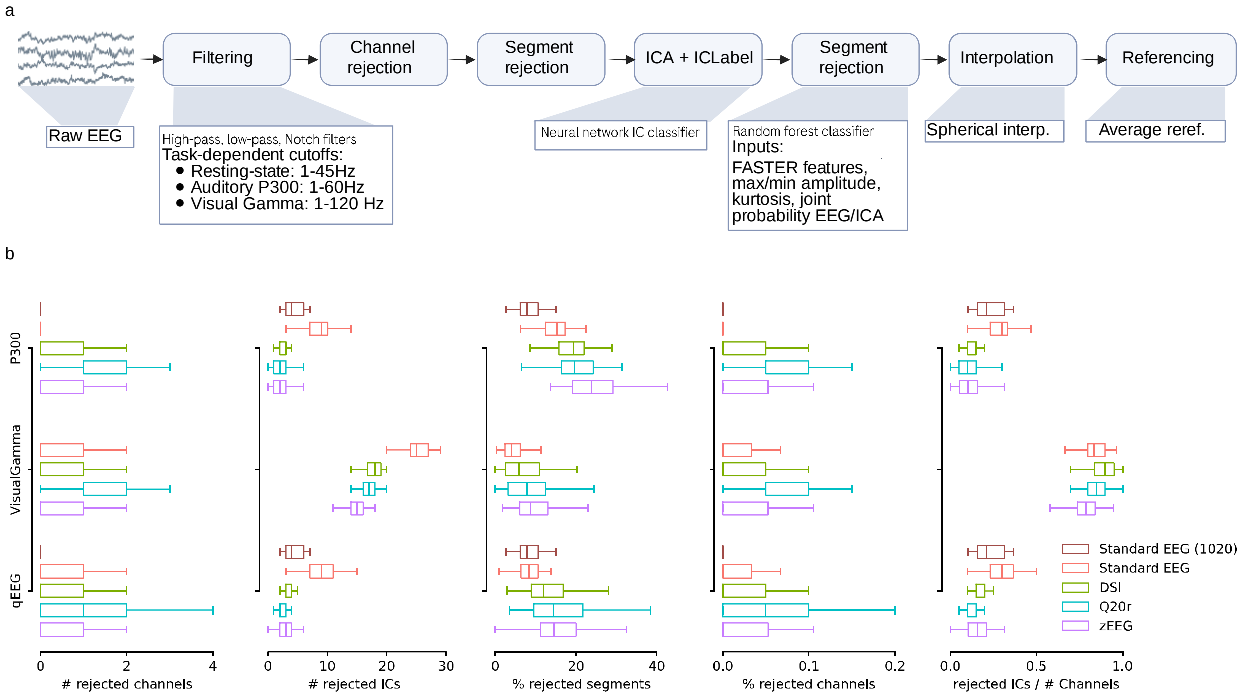

Fig. S1 Preprocessing workflow and statistics across devices and tasks. (a) Steps in the automated preprocessing pipelines applied across tasks. (b) Rejection statistics across tasks and devices resulting from the automated preprocessing pipeline. Please note that for the visual gamma task, all analyses used a subset of channels present in both Standard EEG and the standard 1020 montage. Therefore, only the standard EEG device was preprocessed.

We employed an automated preprocessing pipeline for consistency in cleaning data for all devices (**Fig. S1a**). The intention was to more objectively benchmark the biomarker utility of all devices after common sense preprocessing has been applied in comparable ways to all of them, rather than after customized and differential preprocessing with custom properties per device.

During preprocessing a pattern emerged where the Standard EEG had fewer rejected channels, more rejected independent components (ICs) and fewer rejected sample periods (**Fig. S1b**). These findings may reflect the larger channel count for the standard EEG, allowing for more ICs to capture noise related signals, as well as an increased susceptibility to noise for the dry-electrode EEG devices (see below). Please note that the segment rejection steps were omitted for the artifact task (see below), since for this particular task the artifactual activity was the main interest.

Probing the susceptibility to noise with instructed artifact conditions

In order to assess the susceptibility of the different EEG devices to different types of noise sources commonly encountered during EEG recordings, we designed an additional artifact task where we recorded EEG during instructed patterns of movement (**Fig. S2, see Supplementary Materials Study Protocol** for details).
Participants were asked to perform an eyes-open resting state first and to then perform eye blinks, large saccadic eye movements, jaw clenching and different types of head movements.

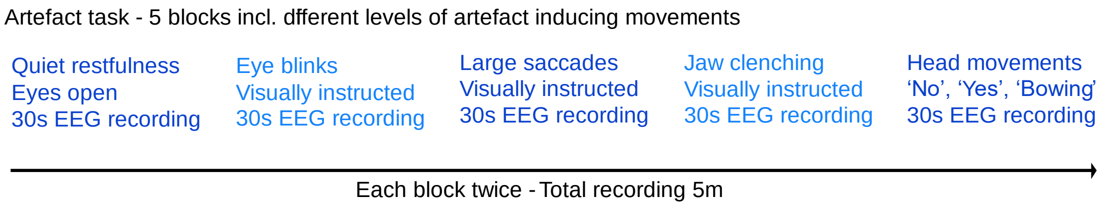

Fig. S2 Experimental design for the artifact task. EEG was recorded while participants went through five task conditions, prompting them to exhibit different degrees of movement types, commonly known to induce artifacts in the EEG. As a low-noise reference, the sequence of task conditions began with an eyes open resting state condition according to the resting state task procedures (**see. Fig. 2**). The resting state recording was followed by visually instructed blinks and horizontal as well as vertical saccades, jaw clenching and lastly large whole head movements. The head movements included three different conditions with rotational movements around the yaw axis (a 'No' gesture), rotational movements around the pitch axis (a 'Yes' gesture) and forth and back movements with the entire upper torso, including the head ('Bowing'). The sequence of task conditions was repeated once, leading to two recordings per condition.

We employed the same automated preprocessing steps as for all other EEG data in this study. However, the segment rejection step was omitted, since the remaining artifactual data was of primary interest.

We computed the average power spectral densities for the different artifact conditions (**Fig. S3**). A consistent pattern emerged where the Saccades condition was most similar to the resting recording for all EEG devices. The conditions blinks, jaw clenching and head movements led to successively increasing broad band offsets in the power spectra, suggesting a gradual increase in artifactual activity across the conditions. The standard EEG showed the smallest differences between all artifact conditions, while all dry-electrode EEG devices showed marked increases in signal, especially for the head movement condition. These observations are consistent with the idea that in particular participant movement may induce much stronger noise in dry-electrode systems than the standard EEG, which may best be able to attenuate movement related signals.

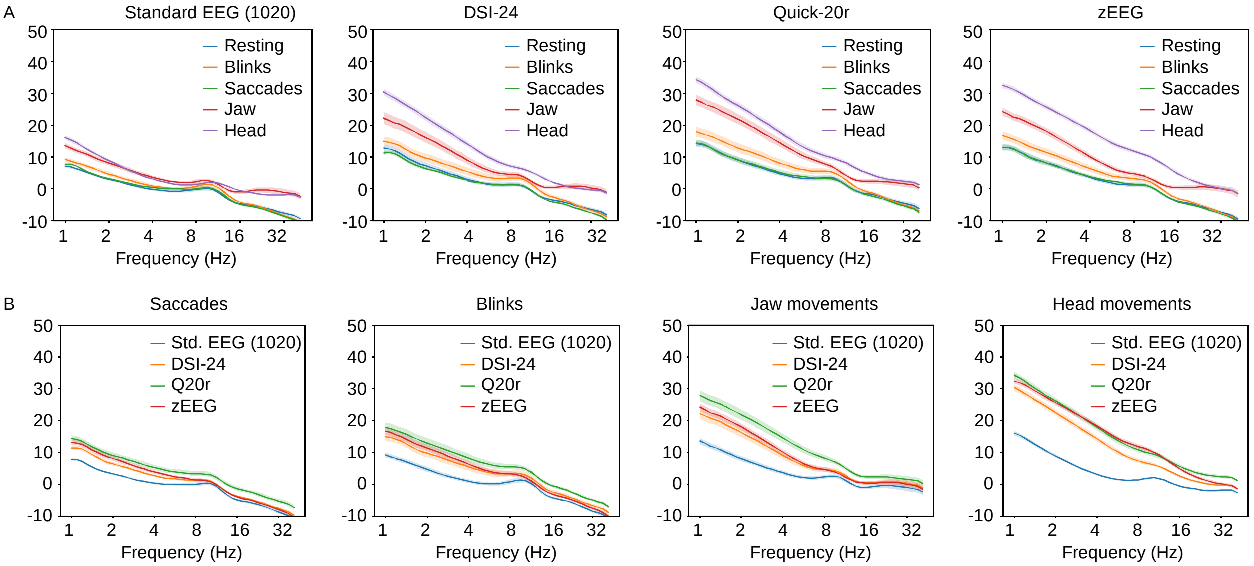

Fig. S3 Average power spectra per artifact condition and per EEG device. (A) Average power spectral densities for each artifact condition grouped by the different EEG devices. The error bars show the 95% confidence interval. (B) Same data as in A grouped by task condition. The error bars show the 95% confidence interval.

We next quantified the absolute agreement and consistency of power features for each device and artifact condition. We compared each artifact condition to the resting state recording done with the same device (**Fig. S4 A&B**) as well as to the standard EEG during the same artifact condition (**Fig. S4 C&D**).

Consistent with the pattern for the power spectra, the Saccades conditions was most comparable to the resting state recordings for each device with moderate to excellent ICC values. The standard EEG was also able to maintain relatively high ICC values during the Blinks condition, while the dry-electrode devices were more affected.

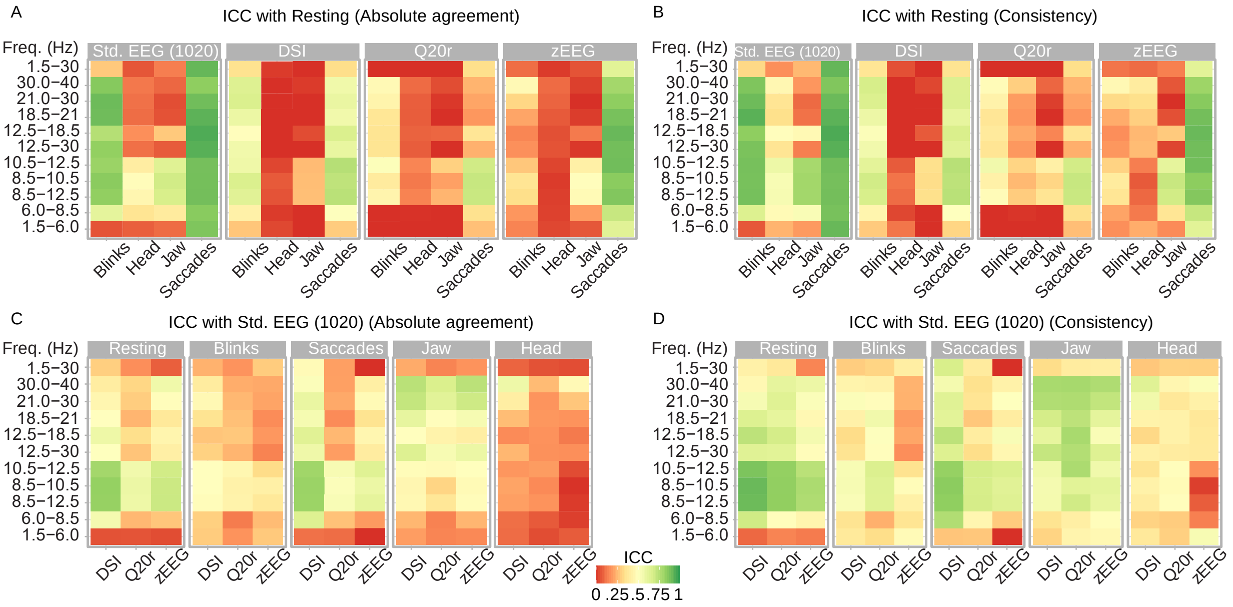

Fig. S4 Agreement and consistency of EEG power features as compared to the resting state condition and the standard EEG. (A) Intraclass correlations (ICC) for the absolute agreement of power features for each device, comparing the different movement conditions to the eyes-open resting state condition recorded at the beginning of the artifact task. (B) Same as A but for the consistency between resting state and movement conditions. (C) Absolute agreement between the standard EEG and the dry-electrode EEG devices for each different task condition. (D) Same as A but for the consistency between standard EEG and the dry-electrode EEG devices.

The jaw-clenching and head movements conditions most severely deteriorated the comparability of the power features to the resting recording, with higher (>12.5 Hz) and lower (<6 Hz) activity being affected most and alpha activity (6-12.5 Hz) being least affected.
Consistent with the above observations, the saccades condition and alpha activity remained most comparable between the dry-electrode EEG devices and the standard EEG, while the other conditions successively degraded the comparability to the standard EEG with the head movements conditions having the strongest effect. Interestingly, the jaw clenching condition led to an increase in ICC values between the standard EEG and dry-electrode devices for higher frequency activity (>12.5Hz), consistent with the observation that muscle related artifacts typically consist of large amplitude, high-frequency activity that consistently appears to affect all EEG devices in the same way. A complete separation of brain-derived from peripheral signals is not possible in most EEG settings. Thus, the observation of consistent noise signals demonstrates a caveat in the interpretation of the ICC values. High consistency between devices may not only reflect consistent access to brain physiology but also consistent noise issues with recordings as well.

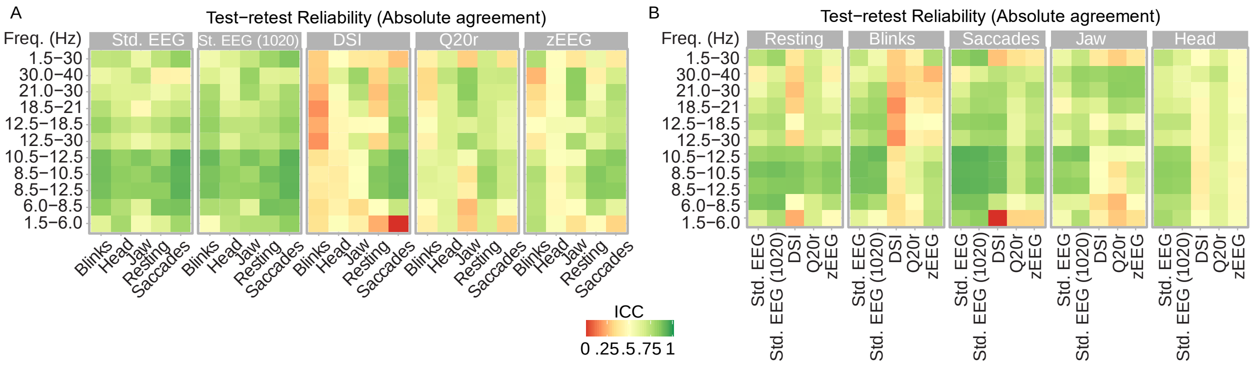

Fig. S5 Test-retest reliability for devices and the task conditions. (A) Intraclass correlations (ICC) for the absolute agreement of spectral power features between Day 1 and Day 8 shown for each movement task condition and grouped by EEG devices. (B) Identical data as in A but grouped by task conditions.

Lastly, we computed the test-retest reliabilities for all artifact conditions and devices (**Fig. S5**). We observed mostly moderate to excellent test-retest reliability for the standard EEG with reduced reliability for the dry-electrode devices that depended on the condition. Here it again is important to emphasize that it is not possible to clearly assign whether the retest-reliabilities are mainly driven through neuronal signals or consistency in the noise sources with the latter being more likely. However, for all devices the highest reliability was observed in the alpha range, which typically is the feature with strong activity contributions from brain sources for resting state EEG recordings.
