# Supplementary material for: Benchmarking the utility of dry-electrode electroencephalography for clinical trials
Source: Sci Rep. 2025 Sep 29;15:33667. doi: 10.1038/s41598-025-18184-7 (PMC12480468; doi:10.1038/s41598-025-18184-7)
Supplement: Supplementary file 2 — Supplementary Information 2. [file 41598_2025_18184_MOESM2_ESM.pdf]

Supplementary information – Study protocol

Benchmarking the utility of dry electrode Electroencephalography for clinical trials

Joseph Paillard*[1], Philipp Bomatter*[1,2], Laura Dubreuil-Vall[1,3], Jörg F. Hipp[1], David J. Hawellek[1&]

1. SYNOPSIS

Name of Company:

Title of Study:

EEG Device Testing Protocol

Principal/Coordinating Investigator:

Study center:

The study will be conducted at

Clinical Phase:

Non-interventional study in healthy volunteers

Rationale:

Electroencephalogram (EEG) assessments in clinical trials are routinely performed using non-invasive, wet scalp electrodes. Preparing the wet EEG cap is a time-consuming procedure that can be burdensome to study participant.

New EEG recording devices using dry or semi-dry electrodes may substantially reduce the set up time and burden for patients and clinical sites. However, the signal quality and comparability of recently developed EEG systems to the current standard wet EEG system are unclear.

The main objective of this study is to compare the quantitative performance of three EEG recording devices in terms of signal quality, their ease-of-use through clinical sites and participant's comfort, versus the standard set-up of a wet EEG cap.

The objective of the study is not to perform any absolute assessment of the performance of the devices (each device has already been certified and is considered as fully functional). The purpose of this project is to compare each tested device to a reference EEG system.

Objective(s) and associated endpoints:

	**Objective**	**Endpoint**
**Primary**	To compare the quantitative performance in terms of signal quality between three new EEG systems and a standard clinical wet EEG system	- EEG features derived from resting state recordings<br>- Latencies and amplitudes of evoked components as well as Signal-to-Noise Ratio from an auditory oddball task<br>- Visual gamma activity (e.g. power and peak frequency)<br>- Test-retest reliability of EEG derived features during resting state and task recordings<br>- Robustness to artefacts from non-neural sources
Secondary	To compare the participant burden between three new EEG systems and a standard clinical EEG system	- Subjective comfort assessed at multiple time points during a recording session<br>- Interim debrief obtained after recording with each device for detailed feedback on each device<br>- Exit debrief at the end of a recording day including a subjective rank ordering of devices from best to worst

Secondary	To compare the site (Technician) burden between three new EEG systems and a standard clinical EEG system	- Set-up (from installation of the system to start of the recording) and clean-up timings for each EEG device<br>- Interim debriefs on ease of clean up<br>- Exit debrief at the end of a recording day including a subjective rank ordering of devices for the best and the worse one<br>- Exit debrief at the end of the study including a subjective rank ordering of devices for the best and the worse one as well as some explanation of the selection

Design:

This study will be performed at a single center, in a Phase 1 unit and involve neurotypical female and male participants. The experimental environment is intended to mimic closely the conditions of a clinical trial, in order to allow for the identification of the ideal EEG system for future clinical trials.

The study will consist of two assessment visits per participant at the clinical site (initial test sessions at D1 Visit 1 followed by re-test sessions at D8 Visit 2, approximately one week later).

The participants will be informed of study specific lifestyle restrictions in a phone call before the first visit.

The staff will be trained on the study conduct, including dummy runs, before the first visit of the first participant.

At each assessment visit, each participant will perform EEG recordings using the 4 devices (test device 1, test device 2, test device 3 and the reference wet EEG cap) and each device session will consist of 4 separate EEG recording types: resting state, auditory oddball task (P300), visually stimulation (specifically targeting activity in the γ band), a controlled artefact session (investigating the sensitivity of the systems to a series of standardized actions known to generate EEG interferences). Ease-of-use and comfort questionnaires will be completed after each test session by the participant as well as the technician.

The sequence of EEG devices recorded within each participant will be randomized according to a Latin square design, and consecutive EEG recordings will be separated by half an hour.

The same order of EEG devices will be applied to both visits of each participant to minimize within participant variability.

Number of participants:

A total of 32 neurotypical male / female participants will be included:

The ratio of female participants should be approximately 50%, to the extent possible.

Rationale for Number of Participants:

No sample size estimation was performed for this study. The number of participants is considered to be sufficient to reach the objectives of the study.

Number of study centers:

1

Duration of the study:

The duration of study participation by the participants will be approximately one week.

Study participation will consist of two one-day clinic visits, approximately one week apart.

Medical Devices:

**Standard EEG kit:**	The device set-up comprises an EEG cap; EEG signals will be recorded using [standard EEG].
**Test Device 1:**	The data from the standard EEG kit will be compared with the one recorded on the [Test Device 1].
**Test Device 2:**	The data from the standard EEG kit will be compared with the one recorded on the [Test Device 2]
**Test Device 3:**	The data from the standard EEG kit will be compared with the one recorded on the [Test Device 3]

Diagnosis and main criteria for inclusion / exclusion:

Inclusion:

Participants must satisfy all of the following inclusion criteria before being allowed to enter the study:

1. A signed and dated informed consent form before any study-specific screening procedure is performed,
2. Aged 18 to 45 year-old (inclusive),
3. Able to undergo study assessments and willingness to comply with study procedures.
4. Within the limit of allowed head circumferences (between 54 and 60 cm)

Exclusion:

If any of the following exclusion criteria apply, the participant must not enter/continue in the study:

1. Any hair style incompatible with the proper wearing of an EEG cap, which is required for good quality EEG recording;
2. A history of any neurological or psychiatric disorder;
3. Participation in an investigational drug or device study within 4 weeks, or five times the half-life (if it is a drug study) of the investigational molecule (whichever is longer), prior to the clinic visit. The participant must not take part in any other trial during the study;
4. Significant changes in the consumption of alcohol, caffeine or xanthine containing products within 48 hours prior to each visit of the study
5. CNS-active medications within 4 weeks prior to the study,
6. Positive test for drugs of abuse or alcohol and/or substance abuse/dependence during the last 12 months;

Prior / Concomitant Medications and Study Lifestyle Restrictions:

No CNS-active medications are allowed during and 4 weeks prior to the study. Participants will be instructed to refrain from strenuous physical activity one day before and on the day of the clinic visit and to maintain a stable routine, compliant with the inclusion and exclusion criteria.

Study Schedule:

Screening:

The participants will be called two weeks before the D1 Visit 1 and be informed of lifestyle restrictions prior to study inclusion.

Participants will be evaluated for eligibility on the Day 1 Visit 1 of the on-site assessments, prior to their potential inclusion in the study.

Written informed consent and if applicable, minor’s assent, will be obtained before any study procedure is performed. The clinic visit will consist of:

- Review of the inclusion/exclusion criteria,

Recording of lifestyle habits: prior alcohol/coffee/stimulant drink consumption, sleeping habits/duration

- Abbreviated physical examination,
- Alcohol breath test,
- Urine drug screen.

**<u>Study periods:</u>**<br><br>***Assessment visit: (D1 Visit 1 and D8 Visit 2)***<br><br>- Screening (D1 Visit 1 only) and eligibility check<br>resting state EEG, sensory stimulation tests (auditory and visual tasks), and controlled artefact stimulation test;<br><br>- Clinical staff questionnaires;<br>- Participants’ questionnaires,	
**Criteria for evaluation:**	
Assessment visit	***Resting state EEG:*** Absolute and relative spectral power, Connectivity measures based on phase correlations (e.g. wPLI) or amplitude correlations (e.g. orhtogonalized power envelope correlations)<br>***P300 ERP:*** Latencies and amplitudes of evoked components, SNR;<br>***Visual gamma-band stimulation ERP:*** power and peak frequency;<br>***Controlled artefact stimulation test:*** Absolute and relative spectral power, RMS signal, amount of variance explained by artefact components.<br>***Data quality:*** For all recordings the amount of artefact free data that can be used for neurophysiological assessments will be quantified and compared.<br>*Other data collection:* participants’ and staff questionnaires’ answers.
**Statistical methods**	
	The comparability of devices will be assessed using univariate statistics to test for significant differences in derived features (e.g. absolute power) as well as appropriate statistics to assess comparability and stability (test-retest) questions (e.g. correlational statistics, intra-class correlations) where appropriate. Similar methods will be applied to questionnaire type data to generate an overall description of the comparability of device quality, comfort and ease of use in a multiparametric fashion

2. SCHEDULE OF ACTIVITIES (SOA)

	Study Information Call	Visit 1		Visit 2
		D1		D8
		Screening Procedures	Post-inclusion Assessments	Re-test
Early call to inform volunteer about the study and on lifestyle restrictions before D1 Visit 1	X			
Informed Consent Form / Assent Form		X		
Inclusion/ Exclusion Review		X		
Lifestyle entry questionnaire		X[b]		X[b]
Abbreviated Physical Examination		X		
Alcohol Breath Test		X		X
Urine Drug Screen		X		X
Randomization			X[a]	
4 test sessions[c]: resting state EEG, ERP tests (auditory and visual task), and controlled artefact stimulation test			X	X
Participant's questionnaires[e]			X	X
Clinical staff questionnaires [f] [g]			X	X

[a] Randomization;

[b] The participants will be asked if they maintained a stable lifestyle before inclusion (physical activity, hours of sleep).

[c] The device assessment visit consists of 4 sessions, one per device Each session lasts approximately 1 hour and consists of a resting state EEG, ERP tests (auditory oddball and visual ERP), and controlled artefact stimulation test; The order for performing the tasks will be the same for all devices.

[d] The participant's questionnaires will comprise an entry questionnaire, an exit questionnaire (for D1 Visit 1 and D8 Visit 2) and three interim questionnaires: one performed before, between and after (for comfort rating) each task for each device and a third one after a device has been tested and before the next device is set up. A summary description of the timing of questionnaires is provided in Figure P1.

[e] The clinical staff's questionnaires will comprise an entry questionnaire (for D1 Visit 1 only), an exit questionnaire and two interim questionnaires (one for rating the system set-up, and a second one rating device cleaning). A summary description of the timing of questionnaires is provided in Figure P1.

[f] A post-training to equipment questionnaire will be added for the clinical staff to evaluate their expectations in the system.

Figure P1: Timing of Questionnaires on Testing Days

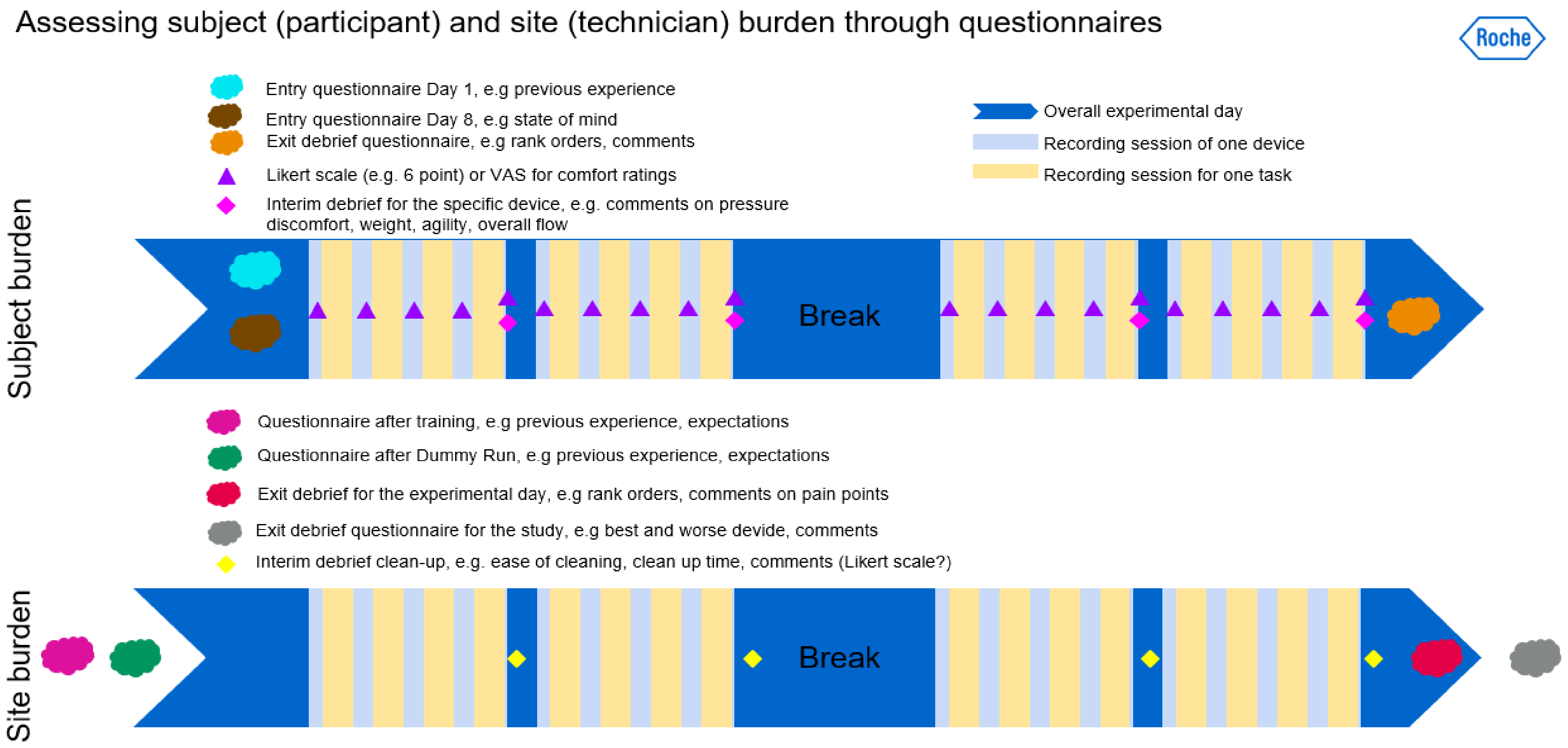

Detailed questionnaires

Subject burden

Entry questionnaire Day 1, e.g previous experience
- Have you previously participated in EEG experiments?
- Have you previously made EEG recordings yourself?
- Have you previously participated in neuroimaging experiments?
- Did you have a tea/coffee today before coming?

Entry questionnaire Day 8, e.g previous experience
- Did you have a tea/coffee today before coming?

Exit debrief questionnaire, e.g rank orders, comments
- Which device would you classify as your preferred one ? And why ?
- Which device would you classify as your less preferred ? And why ?
- What did you observe in terms of comfort?
- What did you observe in terms of ease of use?

Likert scale (e.g. 6 point) or VAS for comfort ratings
- Indicate on a scale from 1 = did not notice it at all to 7 = extremely uncomfortable

Interim debrief for the specific device, e.g. comments on pressure discomfort, weight, agility, overall flow
- How strongly did tactile sensations of the device attract your attention during the recording?
- How strongly did the device feel like a foreign object?
- How strong was your urge to remove the device from your head?

Site burden

Questionnaire after training, e.g previous experience, expectations
- Years of experience in performing EEG experiments
- Previous experience with new EEG devices (active electrodes, wireless, recording software)
- What are your expectations?

Questionnaire after the Dummy Runonce, e.g expectations
- Which device would you classify as your preferred one ? And why ?
- Which device would you classify as your less preferred ? And why ?

Exit debrief for the experimental day, e.g rank orders, comments on pain points
- Please order the devices according to your overall preference from 1 = best device to 4 = worst device
- What did you observe in terms of set up
- What did you observe in terms of clean up
- What did you observe in term sof recordings?

Exit debrief questionnaire for the study e.g best and worse devide, comments
- Which device would you classify as your preferred one ? And why ?
- Which device would you classify as your less preferred ? And why ?

Interim debrief clean-up, e.g. ease of cleaning, clean up time, comments (Likert scale?)
- Indicate on a scale from 1 = very easy to clean up to 7 = extremely difficult to cleanup

LIST OF ABBREVIATIONS

AASP	Adolescent/Adult Sensory Profile
ANOVA	Analysis of Variance
CRO	Contract Research Organization
CV	Coefficient of Variation
D	Day
EEG	Electroencephalogram
EOG	Electrooculogram
ERP	Event-related-potential
ET	Eye tracking
FU	Follow-up visit
HEOG	Horizontal EOG
HEOGL	Left HEOG
HEOGR	Right HEOG
ICF	Informed Consent Form
ICH	International Conference on Harmonization
IEC	Independent Ethics Committee
SOA	Schedule Of Activities
VEOG	Vertical EOG
VEOGL	Upper VEOG
VEOGU	Lower VEOG
WHODRUG	World Health Organization Drug Dictionary
WMA	World Medical Association

3. INTRODUCTION

Electroencephalogram (EEG) assessments in clinical trials are performed using non-invasive scalp electrodes. These electrodes require the application of a highly conductive electrolytic EEG gel between the scalp and the electrode to obtain good contact and reduce the impedance of the skin-electrode interface. Preparing the wet EEG cap is a time-consuming procedure that can be burdensome for patients.

New EEG recording devices that use dry or semi-dry electrodes may substantially reduce the set up time and burden for patients and clinical sites, however their signal quality and comparability to the current standard are unclear.

The main objective of this study is to compare the quantitative performance in terms of signal quality of three EEG recording devices, their ease-of-use through clinical sites and study participant’s comfort, versus the standard set-up of a wet EEG cap.

The objective of the study is not to perform any absolute assessment of the performance of the devices (each device has already been certified and is considered as fully functional). The purpose of this project is to compare each tested device to a reference EEG system.

4. OBJECTIVES AND ENDPOINTS

	Objective	Endpoint
**Primary**	To compare the quantitative performance in terms of signal quality between three new EEG systems and a standard clinical wet EEG system	- EEG features derived from resting state recordings<br>- Latencies and amplitudes of evoked components as well as Signal-to-Noise Ratio from an auditory oddball task<br>- Visual gamma activity (e.g. power and peak frequency)<br>- Test-retest reliability of all EEG derived features during resting state and task recordings<br>- Robustness to artefacts and non-neural signal sources
**Secondary**	To compare the participant burden between three new EEG systems and a standard clinical EEG system	- Subjective comfort assessed at multiple time points during a recording session<br>- Interim debrief obtained after recording with each device for detailed feedback on each device<br>- Exit debrief at the end of a recording day including a subjective rank ordering of devices from best to worst<br>- Exit debrief at the end of the study including a subjective rank ordering of devices for the best and the worse one as well as some explanation of the selection
**Secondary**	To compare the site (Technician) burden between three new EEG systems and a standard clinical EEG system	- Set-up (from installation of the system to start of the recording) and clean-up timings for each EEG device<br>- Interim debriefs on ease of clean up<br>- Exit debrief at the end of a recording day including a subjective rank ordering of devices for the best and the worse one<br>- Exit debrief at the end of the study including a subjective rank ordering of devices for the best and the worse one as well as some explanation of the selection

5. STUDY DESIGN

5.1. Overall Design

This is a study that will be performed at a single center involving neurotypical female and male participants.
The study will consist of two assessment visits per participant the clinical site (initial test sessions at D1 Visit 1 followed by re-test sessions at D8 Visit 2, approximately one week later).
The participant will have been informed of study specific lifestyle restriction by a phone call before attending the first visit.
The staff will have to follow a training session on the full equipment including dummy runs followed by a post-training questionnaire before the first visit.
At each assessment visit, each participant will be subjected to EEG recordings using the 4 devices (test device 1, test device 2, test device 3 and the reference wet EEG cap) and each test session will consist of the recording of a resting state EEG followed by an auditory oddball task (P300), by a visually evoked potential test (specifically targeting activity in the γ band), a controlled artefact stimulation test (investigating the sensitivity of the systems to a series of standardized actions known to generate EEG interferences). All questionnaires will be completed after each test session, before the participant can proceed to the next session.

5.2. Number of Participants

A total of thirty-two (32) neurotypical male / female participants will be included.

The ratio of female participants should be approximately 50%, to the extent possible.

5.3. Number of Study Centers

The study will be performed at one (1) investigational site in Rennes (France).

5.4. End of Study Definition and Duration of Participation

The end of the study is defined as the date of the last visit of the last participant.

The maximum duration of the study for a participant will be approximately 1 week between the screening / first test visit (D1 Visit 1) and the re-test visit (D8 Visit 2).

5.5. Identification of Source Data

The source data will consist of the data generated on-site by the EEG, and other assessments, the questionnaires and the data collected in the Investigator's source data book.

6. STUDY POPULATION

Each participant must participate in the informed consent process. Participants will sign and date the informed consent form (ICF) before any procedures specified in this protocol are performed.

6.1. Inclusion criteria

Participants must satisfy all of the following inclusion criteria before being allowed to enter the study:

1. A signed and dated informed consent form before any study-specific screening procedure is performed,
2. Aged 18 to 45 year-old (inclusive),
3. Able to undergo study assessments and willingness to comply with study procedures.
4. Within the limit of allowed head circumferences (between 54 and 60 cm)

6.2. Exclusion criteria

If any of the following exclusion criteria apply, the participant must not enter/continue in the study:

1. Any hair style incompatible with the proper wearing of an EEG cap, which is required for good quality EEG recording;
2. A history of any neurological or psychiatric disorder;
3. Participation in an investigational drug or device study within 4 weeks, or five times the half-life (if it is a drug study) of the investigational molecule (whichever is longer), prior to the clinic visit. The participant must not take part in any other trial during the study;
4. Significant changes in the consumption of alcohol, caffeine, or xanthine-containing products within 48 hours prior to each visit of the study,
5. CNS-active medications within 4 weeks prior to the study,
6. Positive test for drugs of abuse or alcohol and/or substance abuse/dependence during the last 12 months;

6.3. Lifestyle restrictions

Participants will be instructed at the early information call to refrain from strenuous physical activity one day before and on the day of the clinic visit.

The participants will be requested to maintain a stable routine for 2 days until the first visit (D1 Visit 1).

6.4. Screen Failures

Screen failures are defined as participants who consent to participate in the clinical study but are not subsequently included, because they fail to comply with inclusion and exclusion criteria.

Individuals who do not meet the criteria for participation in this study (screen failure) may not be rescreened.

7. INVESTIGATED DEVICES

7.1. Device selection

The reference system chosen is the one mainly used for current clinical trials conducted through [CRO], i.e. the [standard EEG], and therefore provides robust references for comparisons.

Regarding the selection of the three tested devices, landscaping of currently available equipment on the market was performed. The final selection was done based on criteria related to operational performances and data quality as assessed in pilot experiments.

A full technical description, as well as detailed operational instructions will be provided in a study specific user manual, according to each device that is separate from this study protocol.

7.2. Standard EEG device

[Standard EEG description]

7.3. [Test device 1]

[Test device 1 description]

7.4. [Test device 2]

[Test device 2 description]

7.5. [Test device 3]

[Test device 3 description]

7.6. Method of Device Assignment

The participants will be randomized to each sequence according to a (4x4) Williams Latin square design. The EEG device sequences will be as follows:

Table 1: Randomization

Sequence	Session 1	Session 2	Session 3	Session 4
**1**	A	B	C	D
**2**	B	C	D	A
**3**	C	D	A	B
**4**	D	A	B	C

where A, B, C and D are the reference EEG wet cap, and investigated devices 1, 2 and 3, respectively. In case of incomplete groups of 4 participants, unused participant numbers will be discarded.

The participants’ randomization list will be prepared by Biotrial EEG Corelab.

8. PRIOR TREATMENTS

No CNS-active medications are allowed during and 4 weeks prior to the study. Participants will be instructed to refrain from strenuous physical activity one day before and on the day of the clinic visit.

9. DISCONTINUATION CRITERIA AND RELATED PROCEDURES

9.1. Withdrawal/Discontinued criteria

A participant may withdraw their consent at their discretion.

Any participant may be withdrawn from the study at the discretion of the Investigator.

participants withdrawn or discontinued from the study must not be reincluded.

9.2. Replacement of study participants

Participants that do not complete both visits will be replaced.

10. PROCEDURES

10.1. Staff training

Within the month prior to the start of the study, Biotrial EEG Corelab will organize a training session for the staff that will be performing the experimental procedures.

Each member of staff will perform a dummy run and answer a first questionnaire on their expectancies about the equipment, as shown in Figure P1. The output of the dummy run will be assessed by the EEG Corelab referent in terms of compliance with the instructions.

10.2. Investigational schedule

The schedule of assessments is described in Section 2, Schedule Of Activities (SOA).

10.3. Screening Procedure

The participants will be called 2 working days before the D1 Visit 1 and be informed of lifestyle restrictions prior to study inclusion.

On the day of screening, after the informed consent has been obtained as described in the Inclusion Criteria Section 6.1 and Appendix 1, the following information will be collected: medical history, demographic data, including name, gender and age, current lifestyle. Each participant will have an abbreviated physical examination, alcohol breath test and urine drug screen. Compliance with all inclusion and exclusion criteria will be verified.

Any given included participant will be followed by the same member of staff throughout the study.

10.4. Performance Assessment

10.4.1. Assessment Methods and Experimental Schedule

All experimental procedures will be described in full depth in an EEG Manual accompanying this study protocol as separate document to support transparent and reproducible procedures.

Figure P2: Timing of Tests during each session

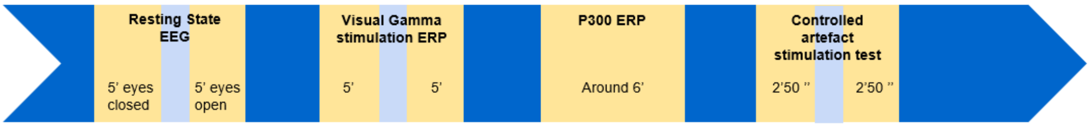

The sessions will have the same order of tasks (as shown in Figure P2) which is:

- Resting State EEG: eyes open and eyes closed
- Visual Gamma-band stimulation
- P300 ERP
- Controlled artefact stimulation test

The overall length of a session for each device is planned to be approx. 2h, including set-up and clean-up time and questionnaire assessments. In between the active recording sessions breaks are planned as well to allow for participants to rest as appropriate., 10

10.4.1.1. **Resting State EEG**

The resting state EEG will be the first record of each session, performed as detailed in the SOA in Section 2. The procedure will overall yield 10-minutes of EEG recording including all EEG channels available on the acquisition system.

The resting-state EEG allows a quantitative measurement of neuronal activity at different frequencies, e.g., power in conventional frequency bands, in the absence of any specific task. Resting-state brain activity will be recorded in two conditions: (1) with eyes closed (5 minutes) and (2) with eyes open (5 minutes). During eyes open condition, participants will be asked to keep looking at a fixed target.

10.4.1.2. **Visual gamma-band stimulation**

Participants are seated with EEG cap mounted and are instructed to sit still and relax. During the task, participants are presented with a visual stimulus consisting of circular, concentric, contracting high contrast sine wave patterns on an external monitor placed at around 69 cm of the participant (annular gratings) following Hoogenboom et al. (10.1016/j.neuroimage.2005.08.043). The annular gratings will have a size of 5 degrees visual angle. The spatial frequency will be 2 cycles per degrees, and the gratings will move at 3 cycles per second, with a contrast of 100%. A red dot will always be shown at the center of the screen for fixation. The participant must keep their gaze at this fixation point and will be asked to refrain from blinking. The stimulus is presented for one second and repeated 300 times between each stimulus, there is a blank period with only the red dot on the screen for a random duration of 1 second to 1.4 second. Spatial frequency and contraction speed are designed to induce reliable visual stimuli in the gamma band. The different grating parameters have been defined according to the publication *Localizing human visual gamma-band activity in frequency, time and space*.

10.4.1.3. **P300 ERP**

Participants are seated with the EEG cap and headphones on and are instructed to sit still and relax and to keep their eyes closed. During the task, participants are being presented with two types of auditory stimuli (“standards” and “deviants”). Both stimuli last 100ms and have the same sound pressure level (85dB) and the same 5ms rise and fall times. The standard and

deviant stimuli have a tone frequency of 500 Hz and 2000 Hz, respectively. For each acquisition the number of deviant stimuli is randomly selected between 25 and 35. The number of standard stimuli is adjusted to have 15% of deviant stimulus frequency. Each interstimulus interval is set randomly and can take values between 1200 and 1900ms. Due to the random number of stimuli the task might last from 5 to 7 minutes (instructions included).
Participants must pay attention to the tones and count the infrequent/deviant tones.

10.4.1.4. **Controlled artefact generation test**

Participants are seated with EEG cap mounted and are instructed to sit still and relax. Prior to the recording, the EEG technician will explain the different actions to be completed during this task. During the task, participants are requested to perform voluntary movements (with the support of real time instructions displayed on the screen) that will generate artefacts in the EEG records. The movements will be following the sequence:

- resting with eyes open for 30 seconds
- eye blinking repeated ten times in 30 seconds when instructed. A cross appears at the screen center when the participant must blink.,
- controlled eye movement: following a moving target on a computer screen (horizontal and vertical saccades),
- jaw tightening and relaxing repeated ten times in 30 seconds when instructed. A cross appears at the screen center when the participant has to clench their jaw.
- three kinds of voluntary head movements, turning their head:
  - from right to left (alternating rotational without moving the chest), a ‘no’ gesture
  - from front to back without moving the chest (low amplitude movements), a ‘yes’ gesture
  - from front to back moving the chest, a ‘bowing’ gesture

All movement condition blocks within the controlled artefact session will be repeated such that two recorded blocks are available per conditions Between the repetition of the 5 movement conditions blocks (30 seconds of resting, eye blinking, controlled eye movements, jaw tightening and head movements), the participant will have one minute to rest freely as they wish.

10.4.2. Outcome measurements

Data metrics to be included in the analysis may include but are not limited to the following.

Resting state EEG

Raw data

- Artefact free ongoing raw EEG activity during eyes open and eyes closed resting state conditions

Derived endpoints

- Absolute power and relative power averaged across artefact free time segments as well as connectivity (phase correlations and amplitude correlations) in different conventional frequency bands (e.g. IPEG guidelines, see table below)

**Band name**	**Delta**	**Theta**	**Alpha**	**Alpha 1**	**Alpha 2**	**Beta**	**Beta 1**	**Beta 2**	**Beta 3**	**Gamma**	**Total**
Lower bound (included) [Hz]	1.5	6	8.5	8.5	10.5	12.5	12.5	18.5	21	30	1.5

Upper bound (excluded) [Hz]	6	8.5	12.5	10.5	12.5	30	18.5	21	30	40	30

Statistical analysis

- Univariate statistics and linear modelling to assess differences in derived endpoints between the test devices and the gold-standard clinical wet system across spectral frequencies.
- Correlational statistics (e.g. intra-class correlation) to assess test-retest reliabilities (D1 to D8) for derived endpoints for each device as well as univariate/resampling statistics to assess differences in test-retest reliabilities between devices.
- Multivariate analyses to decode eyes-closed from eyes-open resting state for each device as well as between the test devices and the gold-standard clinical wet system.

P300 ERP

Raw data

- Time locked EEG responses, for frequent and infrequent tones.

Derived endpoints

- Peak latency and amplitudes of sensory (e.g. N100) and cognitive (e.g. P3b) evoked potentials from the auditory oddball task as well as topographic scalp distributions of the evoked components;

Statistical analysis

- Univariate statistics and linear modelling to assess differences in derived endpoints and scalp topographies between the test devices and the gold-standard clinical wet system.
- Multivariate analyses to decode frequent from infrequent tones for each device as well as a comparison of decodability of frequent and infrequent tones between the test devices and the gold-standard clinical wet system.
- Correlational statistics (e.g. intra-class correlation) to assess test-retest reliabilities (D1 to D8) for derived endpoints and scalp topographies for each device as well as univariate/resampling statistics to assess differences in test-retest reliabilities between devices.

Visual gamma-band stimulation

Raw data

- Time locked EEG responses to the onset and offset of visual grating stimuli during artefact free data epochs.

Derived endpoints

- Time-frequency representations (TFR) of the spectral responses time-locked to the visual stimuli across a set of predefined frequencies (see Resting EEG above)
- Spectral response to visual stimulation in the gamma frequency band based on extant literature (~60-80 Hz)

Statistical analysis

- Univariate statistics and linear modelling to assess differences in derived endpoints and scalp topographies as well as differences in spectral responses between the test devices and the gold-standard clinical wet system.
- Correlational statistics (e.g. intra-class correlation) to assess test-retest reliabilities (D1 to D8) for derived endpoints and scalp topographies as well as spectral responses for each device as well as univariate/resampling statistics to assess differences in test-retest reliabilities between devices.

<u>Controlled artefact stimulation test</u>

<u>Raw data</u>

- Ongoing raw EEG activity segmented into data epochs of predominant artefact types

<u>Derived endpoints</u>

- An analysis treating the artefact segments similar to Resting EEG will be performed (i.e. deriving average signal power and scalp topographies across the spectrum)
- Overall root-mean-square (RMS) signals during artefact periods
- Amount of data remaining after standard artefact cleaning

<u>Statistical analysis</u>

- Univariate statistics and linear modelling to assess differences in derived endpoints and scalp topographies as well as differences in the amount of artefact free data between the test devices and the gold-standard clinical wet system.

10.5. Questionnaires and timing data

10.5.1. Outcome measurements

All questionnaires are provided in Appendix 3 and an overview of the schedule of assessments for the questionnaires is provided in Figure P1.

<u>Participant's questionnaires:</u>

- Entry questionnaire answers;
- Interim-before assessment questionnaire answers;
- Interim-after assessment questionnaire answers;
- Interim-after task questionnaire answers;
- Exit questionnaire answers.

<u>Staff questionnaires:</u>

- Post-training questionnaire answers;
- Interim clean-up questionnaire answers;
- Exit questionnaire at the end of a day answers;
- Exit questionnaire at the end of the study answers.

<u>Set-up and clean-up timing</u>

- Time elapsed between the start of preparing an EEG system to the start of the first recording for each participant
- Time elapsed between the end of a recording session and a fully cleaned experimental set up for each participant

<u>Statistical analysis</u>

For all quantitative questionnaires (VAS) as well as the timing data, univariate statistics and linear modelling to assess differences in ratings between the test devices and the gold-standard clinical wet system will be performed.

Summary overviews of overall preferred rank orders and answers to specific questions will be constructed.

10.5.2. Assessment Methods and Experimental Schedule

Two sets of questionnaires will be used to assess the perception the experimenter and the participants had, of using or being participant to the EEG recording device using VAS as well as open question type assessments (Figure P1).

The experimenter will be asked to rate their expectancy on the equipment, the experimental set-up, the documentation and the level of burden associated with the set-up and cleaning of devices.

The participants will be asked to rate how comfortable they were during the experiment, the clarity of the instructions, and their experience of taking part in these assessments.

The testers will be asked to rate the level of clarity of the active tasks performed as well as the level of burden of the schedule of assessment.

Lastly information on the overall experience with the technology will be gathered.

10.6. Concomitant Medication and Illnesses

10.6.1. Outcome measurements

- Concomitant medication: date and time, name of medication;
- Concomitant illness: date, treatment (if any).

10.6.2. Assessment Methods and Timing

Before a participant leaves the clinical site after the completion of the D1 Visit 1 assessments, the participant will be provided with a diary to collect any concomitant medication or illness during the 4 weeks of remote monitoring.

11. DATA MANAGEMENT AND STATISTICS

11.1. Data entry and management

11.1.1. Data collection

The results from evaluations conducted during the study will be recorded in an appropriate source data book for each participant. All supportive documentation submitted to the Sponsor must be clearly identified with the study or protocol number, and study participant’s number; any personal information, including the study participant’s name, must be removed or rendered illegible to preserve individual confidentiality.

Data from the on-site assessment tests will be collected by Biotrial and transferred to the Sponsor F.Hoffmann-La Roche for further analysis.

All the documents must be archived for a minimum of 15 years or according to the Sponsor’s procedures, whichever is longer.

11.1.2. Data validation

Biotrial will be responsible for the on-site assessment data entry, and their validation (according to the Sponsor requirements).

11.2. **Statistical considerations**

The comparability of devices will be assessed using univariate statistics and linear modelling to test for significant differences in derived endpoints (e.g. absolute power) as well as appropriate statistics to assess comparability and stability (test-retest) questions (e.g. correlational statistics, intra-class correlations) where appropriate. Similar methods will be applied to questionnaire type data to generate an overall description of the comparability of device quality, comfort and ease of use in a multiparametric fashion.

12. REFERENCES

1. Jobert M, Wilson FJ, Ruigt GSF, Brunovsky M, Prichep LS, Drinkenburg WHIM. Guidelines for the recording and evaluation of pharmaco-EEG data in man: The international pharmaco-EEG society (IPEG). Neuropsychobiology 2012; 66: 201-220.

2. Nienke Hoogenboom, Jan-Mathijs Schoffelen, Robert Oostenveld, Laura M. Parkes, and Pascal Fries. Localizing human visual gamma-band activity in frequency, time and space. NeuroImage 29 (2006) 764-773.

3. Connie C. Duncan, Robert J. Barry, John F. Connolly, Catherine Fischer, Patricia T. Michie, Risto Näätänen, John Polich, Ivar Reinvang, Cyma Van Petten. Event-related potentials in clinical research: Guidelines for eliciting, recording, and quantifying mismatch negativity, P300, and N400. Clinical Neurophysiology 120 (2009) 1883–1908

4. W David Hairston, Keith W Whitaker, Anthony J Ries, Jean M Vettel, J Cortney Bradford, Scott E Kerick and Kaleb McDowell. Usability of four commercially-oriented EEG systems. J. Neural Eng. 11 (2014) 046018 (14pp)

13. APPENDICES

Appendix 1: Regulatory and legal considerations

WMA DECLARATION OF HELSINKI – ETHICAL PRINCIPLES FOR MEDICAL RESEARCH INVOLVING HUMAN SUBJECTS

Adopted by the 18th WMA General Assembly, Helsinki, Finland, June 1964
and amended by the:
29th WMA General Assembly, Tokyo, Japan, October 1975
35th WMA General Assembly, Venice, Italy, October 1983
41st WMA General Assembly, Hong Kong, September 1989
48th WMA General Assembly, Somerset West, Republic of South Africa, October 1996
52nd WMA General Assembly, Edinburgh, Scotland, October 2000
53rd WMA General Assembly, Washington DC, USA, October 2002 (Note of Clarification added)
55th WMA General Assembly, Tokyo, Japan, October 2004 (Note of Clarification added)
59th WMA General Assembly, Seoul, Republic of Korea, October 2008
64th WMA General Assembly, Fortaleza, Brazil, October 2013

Preamble

1. The World Medical Association (WMA) has developed the Declaration of Helsinki as a statement of ethical principles for medical research involving human subjects, including research on identifiable human material and data.
The Declaration is intended to be read as a whole and each of its constituent paragraphs should be applied with consideration of all other relevant paragraphs.
2. Consistent with the mandate of the WMA, the Declaration is addressed primarily to physicians. The WMA encourages others who are involved in medical research involving human subjects to adopt these principles.

General Principles

3. The Declaration of Geneva of the WMA binds the physician with the words, “The health of my patient will be my first consideration,” and the International Code of Medical Ethics declares that, “A physician shall act in the patient’s best interest when providing medical care.”
4. It is the duty of the physician to promote and safeguard the health, well-being and rights of patients, including those who are involved in medical research. The physician’s knowledge and conscience are dedicated to the fulfilment of this duty.
5. Medical progress is based on research that ultimately must include studies involving human subjects.
6. The primary purpose of medical research involving human subjects is to understand the causes, development and effects of diseases and improve preventive, diagnostic and therapeutic interventions (methods, procedures and treatments). Even the best proven interventions must be evaluated continually through research for their safety, effectiveness, efficiency, accessibility and quality.
7. Medical research is subject to ethical standards that promote and ensure respect for all human subjects and protect their health and rights.
8. While the primary purpose of medical research is to generate new knowledge, this goal can never take precedence over the rights and interests of individual research subjects.
9. It is the duty of physicians who are involved in medical research to protect the life, health, dignity, integrity, right to self-determination, privacy, and confidentiality of personal

information of research subjects. The responsibility for the protection of research subjects must always rest with the physician or other health care professionals and never with the research subjects, even though they have given consent.

10. Physicians must consider the ethical, legal and regulatory norms and standards for research involving human subjects in their own countries as well as applicable international norms and standards. No national or international ethical, legal or regulatory requirement should reduce or eliminate any of the protections for research subjects set forth in this Declaration.

11. Medical research should be conducted in a manner that minimises possible harm to the environment.

12. Medical research involving human subjects must be conducted only by individuals with the appropriate ethics and scientific education, training and qualifications. Research on patients or healthy volunteers requires the supervision of a competent and appropriately qualified physician or other health care professional.

13. Groups that are underrepresented in medical research should be provided appropriate access to participation in research.

14. Physicians who combine medical research with medical care should involve their patients in research only to the extent that this is justified by its potential preventive, diagnostic or therapeutic value and if the physician has good reason to believe that participation in the research study will not adversely affect the health of the patients who serve as research subjects.

15. Appropriate compensation and treatment for subjects who are harmed as a result of participating in research must be ensured.

Risks, Burdens and Benefits

16. In medical practice and in medical research, most interventions involve risks and burdens.

Medical research involving human subjects may only be conducted if the importance of the objective outweighs the risks and burdens to the research subjects.

17. All medical research involving human subjects must be preceded by careful assessment of predictable risks and burdens to the individuals and groups involved in the research in comparison with foreseeable benefits to them and to other individuals or groups affected by the condition under investigation.

Measures to minimise the risks must be implemented. The risks must be continuously monitored, assessed and documented by the researcher.

18. Physicians may not be involved in a research study involving human subjects unless they are confident that the risks have been adequately assessed and can be satisfactorily managed.

When the risks are found to outweigh the potential benefits or when there is conclusive proof of definitive outcomes, physicians must assess whether to continue, modify or immediately stop the study.

Vulnerable Groups and Individuals

19. Some groups and individuals are particularly vulnerable and may have an increased likelihood of being wronged or of incurring additional harm.

All vulnerable groups and individuals should receive specifically considered protection.

20. Medical research with a vulnerable group is only justified if the research is responsive to the health needs or priorities of this group and the research cannot be carried out in a non-vulnerable group. In addition, this group should stand to benefit from the knowledge, practices or interventions that result from the research.

Scientific Requirements and Research Protocols

21. Medical research involving human subjects must conform to generally accepted scientific principles, be based on a thorough knowledge of the scientific literature, other relevant sources of information, and adequate laboratory and, as appropriate, animal experimentation. The welfare of animals used for research must be respected.

22. The design and performance of each research study involving human subjects must be clearly described and justified in a research protocol.

The protocol should contain a statement of the ethical considerations involved and should indicate how the principles in this Declaration have been addressed. The protocol should include information regarding funding, sponsors, institutional affiliations, potential conflicts of interest, incentives for subjects and information regarding provisions for treating and/or compensating subjects who are harmed as a consequence of participation in the research study.

In clinical trials, the protocol must also describe appropriate arrangements for post-trial provisions.

Research Ethics Committees

23. The research protocol must be submitted for consideration, comment, guidance and approval to the concerned research ethics committee before the study begins. This committee must be transparent in its functioning, must be independent of the researcher, the sponsor and any other undue influence and must be duly qualified. It must take into consideration the laws and regulations of the country or countries in which the research is to be performed as well as applicable international norms and standards but these must not be allowed to reduce or eliminate any of the protections for research subjects set forth in this Declaration.

The committee must have the right to monitor ongoing studies. The researcher must provide monitoring information to the committee, especially information about any serious adverse events. No amendment to the protocol may be made without consideration and approval by the committee. After the end of the study, the researchers must submit a final report to the committee containing a summary of the study's findings and conclusions.

Privacy and Confidentiality

24. Every precaution must be taken to protect the privacy of research subjects and the confidentiality of their personal information.

Informed Consent

25. Participation by individuals capable of giving informed consent as subjects in medical research must be voluntary. Although it may be appropriate to consult family members or community leaders, no individual capable of giving informed consent may be enrolled in a research study unless he or she freely agrees.

26. In medical research involving human subjects capable of giving informed consent, each potential subject must be adequately informed of the aims, methods, sources of funding, any possible conflicts of interest, institutional affiliations of the researcher, the anticipated benefits and potential risks of the study and the discomfort it may entail, post-study provisions and any other relevant aspects of the study. The potential subject must be informed of the right to refuse to participate in the study or to withdraw consent to participate at any time without reprisal. Special attention should be given to the specific information needs of individual potential subjects as well as to the methods used to deliver the information.

After ensuring that the potential subject has understood the information, the physician or another appropriately qualified individual must then seek the potential subject's freely-given

informed consent, preferably in writing. If the consent cannot be expressed in writing, the non-written consent must be formally documented and witnessed.
All medical research subjects should be given the option of being informed about the general outcome and results of the study.
27. When seeking informed consent for participation in a research study the physician must be particularly cautious if the potential subject is in a dependent relationship with the physician or may consent under duress. In such situations the informed consent must be sought by an appropriately qualified individual who is completely independent of this relationship.
28. For a potential research subject who is incapable of giving informed consent, the physician must seek informed consent from the legally authorised representative. These individuals must not be included in a research study that has no likelihood of benefit for them unless it is intended to promote the health of the group represented by the potential subject, the research cannot instead be performed with persons capable of providing informed consent, and the research entails only minimal risk and minimal burden.
29. When a potential research subject who is deemed incapable of giving informed consent is able to give assent to decisions about participation in research, the physician must seek that assent in addition to the consent of the legally authorised representative. The potential subject's dissent should be respected.
30. Research involving subjects who are physically or mentally incapable of giving consent, for example, unconscious patients, may be done only if the physical or mental condition that prevents giving informed consent is a necessary characteristic of the research group. In such circumstances the physician must seek informed consent from the legally authorised representative. If no such representative is available and if the research cannot be delayed, the study may proceed without informed consent provided that the specific reasons for involving subjects with a condition that renders them unable to give informed consent have been stated in the research protocol and the study has been approved by a research ethics committee. Consent to remain in the research must be obtained as soon as possible from the subject or a legally authorised representative.
31. The physician must fully inform the patient which aspects of their care are related to the research. The refusal of a patient to participate in a study or the patient's decision to withdraw from the study must never adversely affect the patient-physician relationship.
32. For medical research using identifiable human material or data, such as research on material or data contained in biobanks or similar repositories, physicians must seek informed consent for its collection, storage and/or reuse. There may be exceptional situations where consent would be impossible or impracticable to obtain for such research. In such situations the research may be done only after consideration and approval of a research ethics committee.

Use of Placebo

33. The benefits, risks, burdens and effectiveness of a new intervention must be tested against those of the best proven intervention(s), except in the following circumstances:
Where no proven intervention exists, the use of placebo, or no intervention, is acceptable; or
Where for compelling and scientifically sound methodological reasons the use of any intervention less effective than the best proven one, the use of placebo, or no intervention is necessary to determine the efficacy or safety of an intervention
and the patients who receive any intervention less effective than the best proven one, placebo, or no intervention will not be subject to additional risks of serious or irreversible harm as a result of not receiving the best proven intervention.
Extreme care must be taken to avoid abuse of this option.

Post-Trial Provisions

34. In advance of a clinical trial, sponsors, researchers and host country governments should make provisions for post-trial access for all subjects who still need an intervention identified as beneficial in the trial. This information must also be disclosed to subjects during the informed consent process.

Research Registration and Publication and Dissemination of Results

35. Every research study involving human subjects must be registered in a publicly accessible database before recruitment of the first subject.

36. Researchers, authors, sponsors, editors and publishers all have ethical obligations with regard to the publication and dissemination of the results of research. Researchers have a duty to make publicly available the results of their research on human subjects and are accountable for the completeness and accuracy of their reports. All parties should adhere to accepted guidelines for ethical reporting. Negative and inconclusive as well as positive results must be published or otherwise made publicly available. Sources of funding, institutional affiliations and conflicts of interest must be declared in the publication. Reports of research not in accordance with the principles of this Declaration should not be accepted for publication.

Unproven Interventions in Clinical Practice

37. In the treatment of an individual patient, where proven interventions do not exist or other known interventions have been ineffective, the physician, after seeking expert advice, with informed consent from the patient or a legally authorised representative, may use an unproven intervention if in the physician’s judgement it offers hope of saving life, re-establishing health or alleviating suffering. This intervention should subsequently be made the object of research, designed to evaluate its safety and efficacy. In all cases, new information must be recorded and, where appropriate, made publicly available.

Appendix 2: Adverse event and serious adverse event definitions

Definitions

- **Adverse Events**

An AE is any untoward medical occurrence in clinical investigation subject.

An AE can therefore be any unfavorable and unintended sign (including an abnormal laboratory finding, for example), symptom, or disease temporally associated with the use of a medicinal product, whether or not related to the medicinal product.

Serious Adverse Events

- **General definitions**

A serious adverse event (SAE) is any untoward medical occurrence that:

- results in death,
- is life-threatening,
- requires inpatient hospitalization or prolongation of existing hospitalization*,
- results in persistent or significant disability/incapacity, or
- is a congenital anomaly/birth defect,
- is an important medical event that requires intervention to prevent one of the above.

** "Inpatient hospitalization" is defined as 24 hours in the hospital or an overnight stay.*

Additionally, important medical events that may not result in death, be life-threatening, or require hospitalization may be considered SAEs when, based on appropriate medical judgment, they may jeopardize the subject and may require medical or surgical intervention to prevent one of the outcomes listed in this definition. Examples of such events include allergic bronchospasm requiring intensive treatment in an emergency room or at home, blood dyscrasias or convulsions that do not result in hospitalization, or development of drug dependency or drug abuse.

Life-threatening refers to immediate risk of death as the event occurred per the reporter. A life-threatening experience does not include an experience that, had it occurred in a more severe form, might have caused death, but as it actually occurred, did not create an immediate risk of death. For example, hepatitis that resolved without evidence of hepatic failure would not be considered life-threatening, even though hepatitis of a more severe nature can be fatal. Similarly, an allergic reaction resulting in angioedema of the face would not be life-threatening, even though angioedema of the larynx, allergic bronchospasm, or anaphylaxis can be fatal.

Hospitalization is official admission to a hospital. Hospitalization or prolongation of hospitalization constitutes criteria for an AE to be serious; however, it is not in itself considered an SAE. In absence of an AE, hospitalization or prolongation of hospitalization should not be reported as an SAE. This is the case in the following situations:

- The hospitalization or prolongation of hospitalization is needed for a procedure required by the protocol.
- The hospitalization or prolongation of hospitalization is part of a routine procedure followed by the center (e.g., stent removal after surgery). This should be recorded in the study file.

In addition, hospitalization for a pre-existing condition that has not worsened does not constitute an SAE.

Disability is defined as a substantial disruption in a person's ability to conduct normal life functions.

If there is any doubt about whether the information constitutes an SAE, the information is treated as an SAE.

Other Reportable Information: Certain information, although not considered an SAE, must be recorded, reported, and followed up as indicated for an SAE. This includes:

- Overdose of an investigational product as specified in this protocol with or without an AE.
- Inadvertent or accidental exposure with or without an AE.

<u>Severity</u>

The maximum intensity of an AE during a day should be graded according to the definitions below and recorded in details. If the intensity of an AE changes over a number of days, then separate entries should be made having distinct onset dates.

1. Mild: AEs are usually transient, requiring no special treatment, and do not interfere with the patient's daily activities.
2. Moderate: AEs typically introduce a low level of inconvenience or concern to the patient and may interfere with daily activities, but are usually ameliorated by simple therapeutic measures.
3. Severe: AEs interrupt a patient's usual daily activity and traditionally require systemic drug therapy or other treatment.

Appendix 3: Questionnaires

Participant Burden

**Participant Burden**	
Entry questionnaire Day 1	• Have you previously participated in clinical trials?<br>○ YES<br>○ NO<br><br>• Have you undergone any EEG examination in the past?<br>○ YES<br>○ NO<br><br>• Have you undergone any brain imaging examination in the past?<br>○ YES<br>○ NO<br><br>• Have you been infected by Covid?<br>○ YES<br>○ NO<br><br>• Did you drink a coffee today before coming?<br>○ YES<br>○ NO<br><br>• Did you drink a tea today before coming?<br>○ YES<br>○ NO<br><br>• Did you drink a glass of alcohol during the last 24h?<br>○ YES<br>○ NO<br><br>• Did you sleep well last night?<br>○ YES<br>○ NO<br><br>• Anything to add? (free comments)
Entry questionnaire Day 8	• Are you happy to come for this second session? And why (free comments)?<br>○ YES<br>○ NO<br><br>• Have you been infected by Covid?<br>○ YES<br>○ NO<br><br>• Did you drink a coffee today before coming?<br>○ YES

	○ NO<br><br>• Did you drink a tea today before coming?<br>○ YES<br>○ NO<br><br>• Did you drink a glass of alcohol during the last 24h?<br>○ YES<br>○ NO<br><br>• Did you sleep well last night?<br>○ YES<br>○ NO<br><br>• Anything to add? (free comments)
Exit debrief questionnaire<br><br>System ranking	Rules (on screen) = Please answer to the next 4 questions, your answers will be used to define your preference list of the systems.<br><br>• Please rank the devices in order of preferences.<br>○ Device #1<br>○ Device #2<br>○ Device #3<br>○ Standard EEG Device<br><br>• Why did you choose your preferred device as the best one?<br><br>• Why did you choose your less preferred device as the worst?<br><br>• Are you looking forward to performing the second session? (Day 1 only) and why (free comments)?<br>○ YES<br>○ NO<br>• Anything to add? (free comments)
After each task<br><br>VAS Comfort	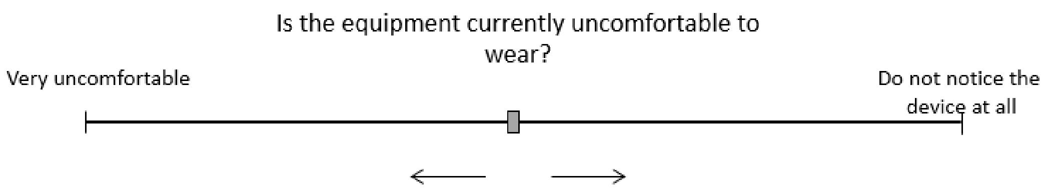
Interim debrief	

VAS tactile sensations	How strongly did tactile sensations of the device attract your attention during the recording? 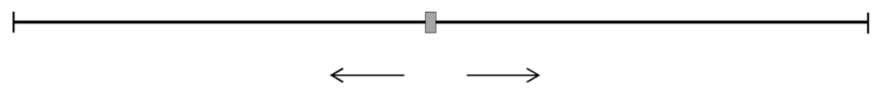
VAS foreign object	How strongly did the device feel like a foreign object? 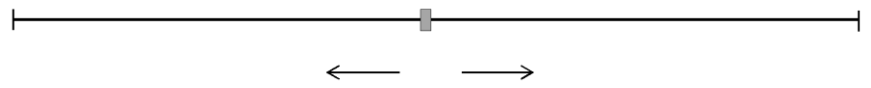
VAS urge to remove	• Do you like the device?<br>○ YES<br>○ NO<br><br>• Anything to add? (free comments)

<u>Site Burden</u>

**Site Burden**	
After training questionnaire	How do you think the new devices can be compared to the reference EEG system in each of the following steps?<br>1: worse<br>2:identical<br>3:better<br>4:much better

	Set-up time participant preparation time	Participant's comfort and acceptability during recording	Clean-up time
Device #1			
Device #2			
Device #3			

• Please rank the devices in order of preferences.

<table>
<tr><td></td><td>
○ Device #1
○ Device #2
○ Device #3
○ Standard EEG Device

• Why did you choose your preferred device as the best one?

• Why did you choose your less preferred device as the worst?

• How many years of experience in running EEG experiments do you have?
○ No previous experience
○ 1 to 5 years
○ 5 to 10 years
○ More than 10 years

• How many years of experience in running clinical trials do you have?
○ No previous experience
○ 1 to 5 years
○ 5 to 10 years
○ More than 10 years

• Do you have a previous experience with the Standard EEG device?
○ YES
○ NO

• Do you have a previous experience with new wireless EEG device?
○ YES
○ NO

• Anything to add? (Free comments)
</td></tr>
<tr><td>After DR (dummy run) questionnaire</td><td>
How do you think the new devices can be compared to the reference EEG system in each of the following steps?
1: worse
2: identical
3: better
4: much better

<table>
<tr><th></th><th>Set-up time participant preparation time</th><th>Participant’s comfort and acceptability</th><th>Clean-up time</th></tr>
<tr><td>Device #1</td><td></td><td></td><td></td></tr>
<tr><td>Device #2</td><td></td><td></td><td></td></tr>
<tr><td>Device #3</td><td></td><td></td><td></td></tr>
</table>
• Please rank the devices in order of preferences.
○ Device #1
○ Device #2
○ Device #3
○ Standard EEG Device
</td></tr>
</table>

	• Why did you choose your preferred device as the best one?<br>• Why did you choose your less preferred device as the worst?<br>• Anything to add? (free comments)
Exit debrief for the experimental day questionnaire	Rules (on screen) = Please answer to the next 4 questions, your answers will be used to define your preference list of the systems<br><br>• Please rank the devices in order of preferences.<br>○ Device #1<br>○ Device #2<br>○ Device #3<br>○ Standard EEG Device<br><br>• Why did you choose your preferred device as the best one?<br>• Why did you choose your less preferred device as the worst?<br>• Anything to add? Please explain if you have changed your ranking (free comments)

Interim debrief clean-up (to place after each session with one system)<br><br>VAS Set-up	• Did the participant have problematic hair?<br>○ YES<br>○ NO<br>○ Why? (free comment)<br><br>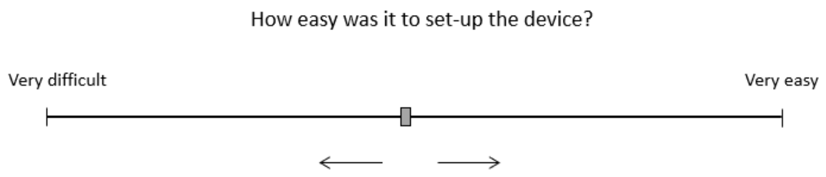<br><br><br>• How long does it take to set-up the device? (answer expected in minutes)<br>• Do you think that the comfort of the participant was negatively impacted by the device?<br>○ YES<br>○ NO<br>○ No opinion
VAS Clean-up	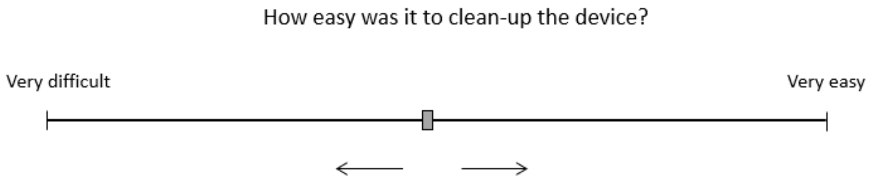<br><br><br>• How long does it take to clean-up the device? (answer expected in minutes)

	• Anything to add? (free comments)

<table>
<tr>
<td>Exit of the study</td>
<td>
Rules (on screen) = Please answer to the next 4 questions, your answers will be used to define your preference list of the systems

• Please rank the devices in order of preferences.
○ Device #1
○ Device #2
○ Device #3
○ Standard EEG Device

• Why did you choose your preferred device as the best one?

• Why did you choose your less preferred device as the worst?

From 1 (worse) to 4 (definitely better), rate the following points in comparison with the standard EEG device
<table>
<tr><td></td><td>Set-up time participant preparation time</td><td>Participant’s comfort and acceptability</td><td>Clean-up time</td></tr>
<tr><td>Device #1</td><td></td><td></td><td></td></tr>
<tr><td>Device #2</td><td></td><td></td><td></td></tr>
<tr><td>Device #3</td><td></td><td></td><td></td></tr>
</table>

• Anything to add? Please explain if you have changed your ranking (free comments)
</td>
</tr>
</table>
